# Single cell multi-omic analysis identifies key genes differentially expressed in innate lymphoid cells from COVID-19 patients

**DOI:** 10.3389/fimmu.2024.1374828

**Published:** 2024-07-04

**Authors:** Abhinav Kaushik, Iris Chang, Xiaorui Han, Ziyuan He, Zsolt I. Komlosi, Xuhuai Ji, Shu Cao, Cezmi A. Akdis, Scott Boyd, Bali Pulendran, Holden T. Maecker, Mark M. Davis, R. Sharon Chinthrajah, Rosemarie H. DeKruyff, Kari C. Nadeau

**Affiliations:** ^1^ Sean N. Parker Center for Allergy and Asthma Research, Department of Pathology, Stanford University School of Medicine, Stanford, CA, United States; ^2^ Department of Genetics, Cell- and Immunobiology, Semmelweis University, Budapest, Hungary; ^3^ Swiss Institute of Allergy and Asthma (SIAF), University of Zurich, Davos, Switzerland; ^4^ Human Immune Monitoring Center, Institute for Immunity, Transplantation, and Infection, Stanford University School of Medicine, Stanford, CA, United States; ^5^ Christine Kühne-Center for Allergy Research and Education, Davos, Switzerland; ^6^ Department of Pathology, Stanford University, Stanford, CA, United States; ^7^ Institute for Immunity, Transplantation and Infection, Stanford University, Stanford, CA, United States; ^8^ Department of Microbiology and Immunology, Stanford University, Stanford, CA, United States; ^9^ Howard Hughes Medical Institute, Stanford University, Stanford, CA, United States

**Keywords:** innate lymphocyte cells (ILCs), SARSCoV- 2, COVID - 19, single cell RNA analysis, single cell immunology

## Abstract

**Introduction:**

Innate lymphoid cells (ILCs) are enriched at mucosal surfaces where they respond rapidly to environmental stimuli and contribute to both tissue inflammation and healing.

**Methods:**

To gain insight into the role of ILCs in the pathology and recovery from COVID-19 infection, we employed a multi-omics approach consisting of Abseq and targeted mRNA sequencing to respectively probe the surface marker expression, transcriptional profile and heterogeneity of ILCs in peripheral blood of patients with COVID-19 compared with healthy controls.

**Results:**

We found that the frequency of ILC1 and ILC2 cells was significantly increased in COVID-19 patients. Moreover, all ILC subsets displayed a significantly higher frequency of CD69-expressing cells, indicating a heightened state of activation. ILC2s from COVID-19 patients had the highest number of significantly differentially expressed (DE) genes. The most notable genes DE in COVID-19 vs healthy participants included a) genes associated with responses to virus infections and b) genes that support ILC self-proliferation, activation and homeostasis. In addition, differential gene regulatory network analysis revealed ILC-specific regulons and their interactions driving the differential gene expression in each ILC.

**Discussion:**

Overall, this study provides mechanistic insights into the characteristics of ILC subsets activated during COVID-19 infection.

## Introduction

The outcome of infection with the severe acute respiratory syndrome coronavirus 2 (SARS-CoV-2) is highly variable. While many patients exhibit mild to moderate symptoms, others progress to severe disease requiring hospitalization, and some to multi-organ failure and death. Many patients recover within a few weeks from coronavirus disease 2019 (COVID-19), while others are known to progress to “long COVID” from which recovery may take months (1, 2). The risk of severe COVID-19 and death in those infected with SARS-COV-2 increases with age and is greater in men than women (1–3). Despite the progress made in vaccine development and treatment for COVID-19, much remains to be elucidated regarding the underlying immune response to COVID-19, and the variability of this response.

Severe COVID-19 is often associated with an activated immune response characterized by enhanced plasma levels of pro-inflammatory mediators including IL-6, IL-1β, TNF, IL-2 and others (2, 4, 5). Whether this is an appropriate response to disease in these patients or a dysregulated immune response, described as “cytokine storm”, is not known. Lymphopenia has often been described in severe COVID-19 patients, including a reduction in CD4^+^ and CD8^+^ T cells and NK cells (6, 7), and an upregulation of exhaustion markers on remaining CD8^+^ T cells and NK cells (8). Dysregulation in other cell types in COVID-19 patients was also reported, including increased numbers of plasmablasts and a novel cell population of developing neutrophils described in COVID patients with acute respiratory distress syndrome (ARDS) (7).

Innate lymphoid cells (ILCs) include ILC1, ILC2, and ILC3 cells which express the α-chain of the IL-7 receptor (CD127) and the developmentally distinct CD127^neg^ NK cells. CD127^+^ ILCs, which are considered innate counterparts of Th1, Th2 and Th17 subsets of CD4^+^ T cells, are largely tissue resident cells and are enriched at barrier surfaces of the mammalian body, such as the lung and intestine, where they respond rapidly to environmental or microbial stimuli, act early in the immune response and contribute to tissue homeostasis and healing. ILCs are also found in peripheral blood, which contains the ILC1 and ILC2 subsets as well as ILC precursor (ILCp) cells (9) which provide a source of ILC1, ILC2 and ILC3 cells as needed by tissues (9–12). Type 1 ILCs (ILC1s) function as a first line of defense against infections with viruses, often in the lung, and Type 2 ILCs (ILC2s) play a key role in lung homeostasis where they have both pathogenic and protective functions. Absolute counts of total ILCs as well as counts of ILC subsets have been reported to be reduced or largely depleted in peripheral blood of patients with COVID-19, particularly in cases of severe COVID-19 (3, 13, 14).

Little is known regarding the role of ILCs in human respiratory virus infections, but studies in mice show that ILC2s accumulate in the lung of virus-infected mice where they may contribute to lung inflammation as well as promote tissue repair (15–17). Since COVID-19 infection is often initiated in the upper airways, we hypothesized that ILCs may play an important role in COVID-19-associated lung inflammation and its subsequent resolution. To our knowledge there have been no systems biology studies probing the transcriptional responses of ILCs at a single cell level in patients with COVID-19, which could provide insight into the role of ILCs in COVID-19 infection.

To more precisely define the impact of COVID-19 on ILCs, we employed a single cell multi-omics approach consisting of Abseq (18) and single-cell RNA sequencing (19) to probe the surface protein marker expression and transcriptional profile respectively, and to evaluate the heterogeneity of CD127^+^ ILCs in peripheral blood of patients with asymptomatic to moderate and long-term COVID-19 compared with healthy control participants. The differential expression analysis revealed that ILCs from COVID-19 patients were transcriptionally distinct from those of healthy control participants.

## Results

### Single-cell transcriptome analysis of ILCs from COVID-19 patients

**Figure 1:**
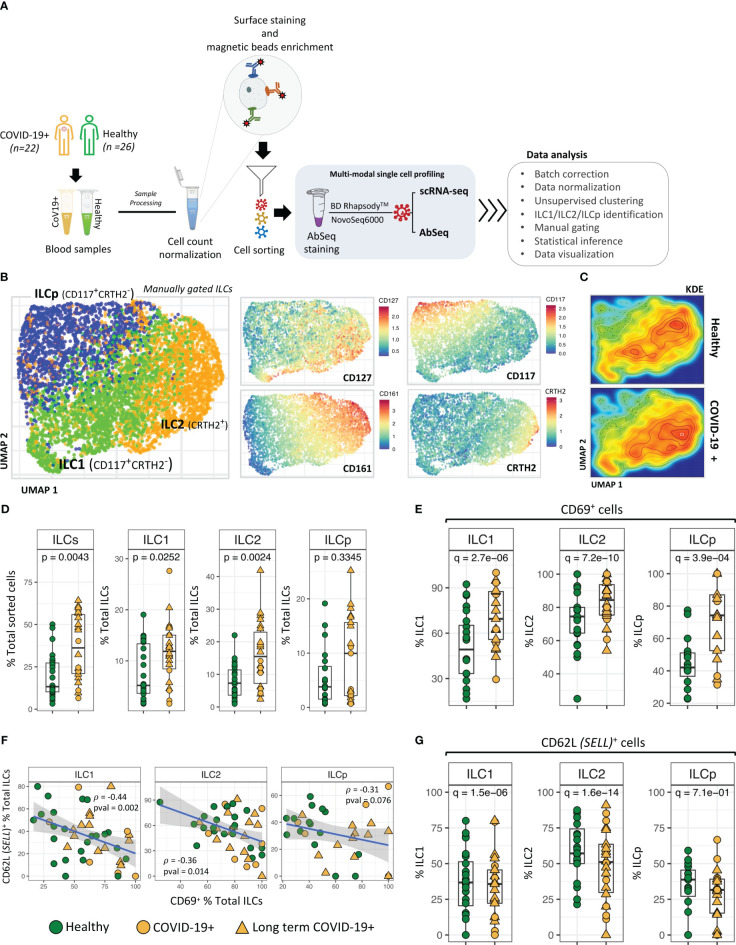
ILC identification and classification. **(A)** Schematic illustrating experimental approach. **(B)** UMAP dimensionality reduction analysis of manually gated ILCs from COVID-19 patients and healthy control participants. ILCs in UMAPs are colored to show the relative expression of CD127, CD161, CD117 and CRTH2. **(C)** Kernel Density Estimation (KDE) of ILC distribution in the UMAPs. The plot represents the density distribution of ILCs from COVID-19 patients and healthy controls. **(D)** Frequencies (i.e., percentage of total live cells) of ILCs and ILC subsets from COVID-19 patients were compared with healthy controls. P-values between two groups of samples were calculated using Wilcoxon rank-sum test. **(E)** Significant differences in CD69 expression by ILC subsets from COVID-19 patients compared with healthy controls. **(F)** Regression analysis showing the inverse association between CD62L^+^ ILCs versus CD69^+^ ILCs in each sample. **(G)** Frequencies (i.e., percentage) of CD62L^+^ ILCs from COVID-19 versus healthy controls. Adjusted p-values between two groups of cells were calculated using Wilcoxon rank-sum test (see methods). For boxplot representation, percentage of cells expressing a given protein in every sample is shown.

We examined peripheral blood ILCs from a total of 22 adult patients with asymptomatic (n=2), mild (n=17), and moderate (n=3) COVID-19, some of whom developed long term COVID-19 assessed at 30 days (n=15) (68.1%) and 90 days (n=12) after infection and 25 healthy control participants (**Supplementary Table S1**). Samples selected for this study were obtained within 76 days of positive COVID PCR test date. For profiling peripheral blood ILCs, we first sorted ILCs from PBMCs, which were then subjected to targeted analysis using BD Rhapsody™ platform (19) (see method). Briefly, we applied simultaneous quantification of surface proteins (Abseq) (18) (**Supplementary Table S2**) and targeted mRNA expression at the single-cell level (**Figure 1A**; **Supplementary Figure 1**). This approach allowed the simultaneous interrogation of surface proteins used for immunophenotyping, immune-related genes and single-cell heterogeneity analysis. From the resulting multi-modal, high-throughput single cell dataset, 6247 ILCs (ILC1 (CD117^-^CRTH2^-^) = 2103; ILC2 (CD117^+/-^ CRTH2^+^) = 2509 and ILCp (CD117^+^CRTH2^-^) = 1635) (**Supplementary Figure 1**), were identified based on the cell surface protein expression profiles using an established ILC manual gating strategy (13, 20). Remaining 10,895 ILCs from sorted cell population were excluded from further analysis.

Unsupervised UMAP based dimension reduction and visualization of all manually gated ILCs (see methods) showed three types of ILCs as distinct cell clusters, corresponding to ILC1, ILC2 and ILCp cells (**Figure 1B**; **Supplementary Figures 2A, B**). As expected, CD127 was expressed by all ILCs, with ILC1s and ILC2s demonstrating high CD127 expression (21). Similarly, we observed a cluster of CRTH2^+^ ILC2 cells, CD161^high^ ILC2 cells and CD117^high^ ILCp cells. In the UMAP based cell clusters, the cell density analysis using Kernel Density Estimation (KDE) of total ILCs from COVID-19 and healthy participants revealed a higher cell density in the ILC2 cluster in COVID-19 patients as compared with healthy participants (**Figure 1C**). We observed relatively higher frequencies of gated ILCs (p=0.004; percentage of total sorted innate lymphoid cells) in peripheral blood of COVID-19 patients as compared to healthy control participants, including a higher frequency of both ILC1 cells (p = 0.007) and ILC2 cells (p = 0.002; **Figure 1D**), also indicated from the kernel density estimation (KDE) heatmaps of ILCs within the UMAP plot (**Figure 1C**). Comparison of LT COVID-19 patients vs non-long-term participants showed relatively higher frequencies of total ILCs (p=0.001) and a trend toward higher frequencies of ILC2 and ILCp in LT COVID-19 patients (**Supplementary Figures 2C, D**). Expression of KLRG1, a marker of a developmentally transitional stage of ILC2 cells, was present in a subset of ILC2 cells and ILCp cells as expected and did not differ significantly between COVID-19 participants and healthy controls (**Supplementary Figures 3A, B**). In addition, we also observed significant increase in the population of CD279 (PD-1)+ ILCs (**Supplementary Figure 3C**) in COVID-19 participants. Interestingly, for ILCps, this significant increase in the proportion of CD279 (PD-1)+ cells is associated with LT COVID-19 participants.

Among the cell surface protein markers used in our Abseq panel, a higher relative frequency of expression of the activation marker CD69 was observed in ILC1 (adj. p = 9.3e-10), ILC2 (adj. p = 6.1e-14) and ILCp (adj. p = 7.9e-5) cells from COVID-19 patients compared with controls. (**Figure 1E**). Intriguingly, the increased activation status was inversely correlated with protein expression of the trafficking molecule CD62 Ligand (CD62L) (**Figures 1F, G**), a marker associated with cell naivety (22) encoded by the gene *SELL*. CD62L protein expression was significantly lower in all three ILC subsets in COVID-19 patients, indicating that these cells were more mature (**Figure 1G**). The significant inverse correlation between CD69 and CD62L was most prominent in ILC1 cells (p = 0.002; ρ = -0.44) as compared to ILC2 (p = 0.014; ρ = -0.36) and ILCp (p = 0.076; ρ = -0.31). Although there was a range of days post COVID-19 RT-PCR positive diagnosis in which peripheral blood was obtained (**Supplementary Figure 4A**), relative frequencies of ILCs expressing CD69 or CD62L were not associated with days since the PCR visit date. (**Supplementary Figures 4B, C** *).*

### Single cell mRNA expression revealed key genes differentially expressed in ILC1, ILC2 and ILCp subsets in COVID-19 patients

**Figure 2:**
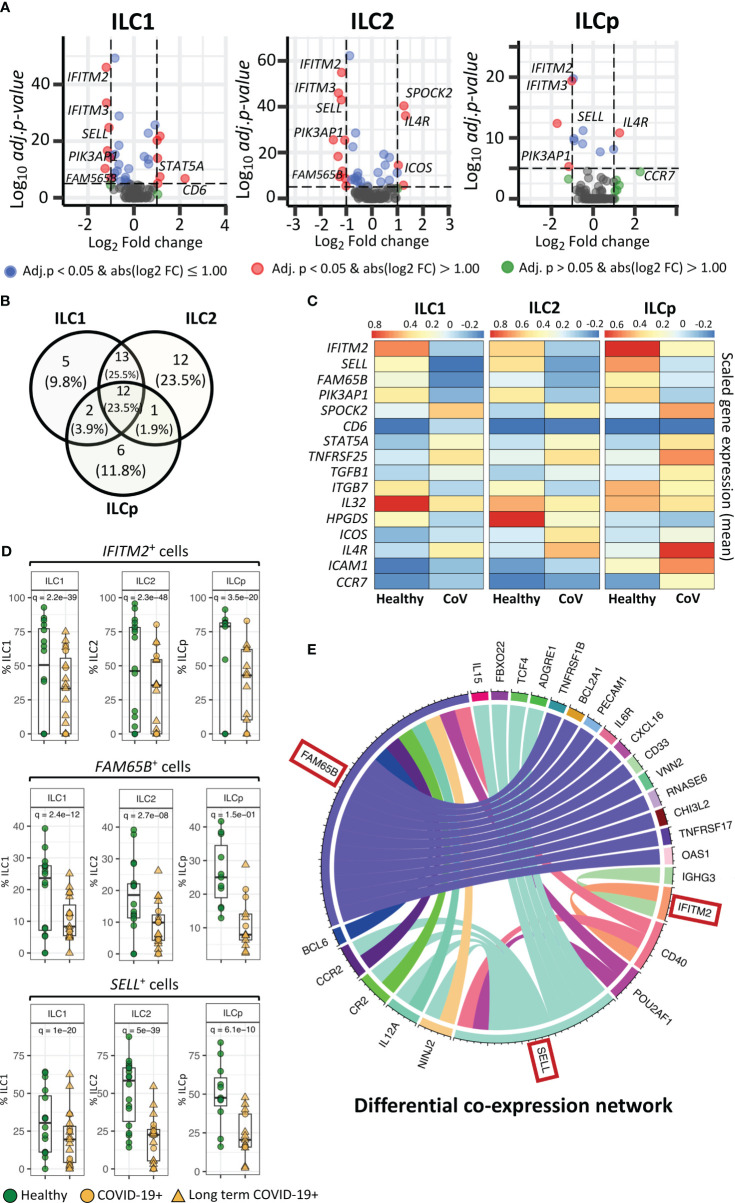
Genes differentially expressed in ILCs from COVID-19 patients compared with healthy controls. **(A)** Volcano plots representing DE transcripts in sorted ILCs from COVID-19 patients and controls. **(B)** Venn diagram showing the number of DE genes in each ILC subset. We observed 13 DE genes common to all three ILC subsets from COVID-19 patients relative to healthy controls. **(C)** Heatmap depicting mean (scaled) expression of selected DE genes in COVID-19 patients and healthy controls in each ILC subset. **(D)** Frequencies (i.e., percentage) of *IFITM2* ^+^, *FM65B* ^+^ and *SELL* ^+^ ILCs from COVID-19 patients versus healthy controls. **(E)**. The circos plots representing differential co-expression networks reconstructed for *IFITM2*, *FM65B* and *SELL* genes in total ILC population. The edges in the plot represents co-expression links significantly different across healthy vs COVID-19 ILCs. We observed that among *IFITM2*, *FM65B* and *SELL* genes*, FAM65* shows highest degree of differentially co-expressed links with other genes. Adjusted p-values for enumerating DE genes between two groups of cells were calculated using MAST test (see methods). For boxplot representation, percentage of cells expressing the given gene in every sample is shown.

We investigated transcriptional differences in ILCs from COVID-19 patients compared with healthy control participants (**Figures 2A, B**). The scRNA-seq was performed using BD Rhapsody multi-modal single cell analysis platform that quantified the expression of 430 genes. However, the number of genes expressed (expression > 0) by the gated ILCs varied between 16 to 108. **Supplementary Table S3** shows median, and range of gene count expressed in different ILC subsets in healthy and COVID-19 individuals. The differential expression analysis revealed a higher number of genes significantly downregulated than upregulated in COVID-19 patients in ILC1, ILC2 and ILCp cells (**Supplementary Figure 5**), including *SELL, IFITM2* and *FAM65B*. Some genes were DE in all three subsets, some genes were DE in two ILC subsets, and other genes were uniquely DE by ILC1s, ILC2s, or ILCps compared with healthy controls, as shown in **Figure 2B**. Overall, ILC2s from COVID-19 patients had the highest number of significantly DE genes (41 genes, log fold-change > 0.5 and adj.p< 0.05), followed by ILC1s (35 genes) and ILCps (19 genes) (**Figure 2B**). ILC1 and ILC2 cells had the highest number of shared DE genes (*n*= 27) in COVID-19 patients compared to healthy participants. ILC2s showed 12 unique DE genes not observed in ILC1 and ILCp cells (**Figure 2B**).

Within the list of significantly DE genes, interferon-induced transmembrane protein 2 (*IFITM2*), *FAM65B* and *SELL* were downregulated in all ILC subsets in COVID-19 patients compared with healthy controls (**Figures 2C, D**). *IFITM2* was downregulated in all ILCs: ILC1s (adj.p = 2.2e-39), ILC2s (adj.p = 2.3e-48) and ILCps (adj.p = 3.5e-20) (**Figures 2A, C, D**). Another member of the *IFIT* family, i.e., *IFITM3*, was also significantly downregulated in all ILC subsets: ILC1s (adj.p = 1.7e-31), ILC2s (adj.p = 8.2e-44) and ILCp (adj.p = 8.7e-20) (**Figure 2A**; **Supplementary Data File 1**). Two other genes, *FAM65B*, which negatively regulates T cell activation, adhesion and migration, and *SELL*, which encodes the trafficking molecule CD62L, were also significantly downregulated in all three ILC subsets of COVID-19 patients compared with controls (**Figure 2C, D**). Interestingly, pathway analysis of DE genes in each ILC reveals several interesting pathways (**Supplementary Figure 6**), including pathways related to T-cell activation/co-stimulation, cell adhesion, immune response regulation and cytokine signaling. Notably, in ILC2, we also observed pathways specific to “T-cell activation in SARS-COV-2” and “Th1 and Th2 cell differentiation”. Whereas, in ILCps, we observed pathways related to leukocyte migration and regulation of cell surface adhesion.

To gain insights if these pathways are associated with patient with LT COVID-19 symptoms, we performed differential gene expression analysis of ILCs from LT COVID patients versus healthy participants (**Supplementary Figure 7A**; **Supplementary Data File 2**). We observed that DE genes in LT COVID patients were similar to those in the comparison of ILCs from all COVID-19 patients with healthy participants (**Supplementary Figure 7B**.). Most of DE genes predicted were conserved across the two different analyses, including the genes *IFITM2*, *SELL*, and *FAM65B*, which were also predicted to be significantly downregulated in LT COVID-19 patients compared with healthy participants. As expected, enrichment analysis of DE genes predicted with LT COVID-19 patients versus healthy participants reveals similar set of pathways related to T-cell activation, cytokine signaling, cell adhesion and migrations (**Supplementary Figure 8**). Interestingly, several pathways were commonly enriched across three ILC subtypes. Two notable pathways highlighted were “Leukocyte activation” and “Positive regulation of immune response”.

In a cellular system, two or more genes can co-express together to form a gene-expression network. Genes that are co-expressed together tend to be involved in the same biological processes or pathways. During perturbation such as COVID-19 infection, the interactions between genes can change as the ILCs responds to the virus, which can alter their functional role. Differential co-expression network analysis allows us to compare the networks of gene interactions in ILCs from COVID-19 patients versus healthy individuals. Highly connected genes within the differential co-expression network represent genes that are likely to play important coordinating roles in the cellular response to COVID-19 infection, as their expression is highly correlated with many other genes only in the COVID-19+ condition. Identifying such genes provides insights into the key drivers of the host response to the virus and potential molecular targets for therapeutic intervention. Therefore, we reconstructed two gene co-expression cellular networks, i.e. COVID-19 ILCs and healthy ILCs, in which each network is composed of pair-wise gene interactions among all the genes available in the total ILCs. Next, we performed differential analysis of two networks and identified group of genes that changed their interactions with three DE genes- *IFITM2*, *FAM65B* and *SELL*, across COVID-19 vs healthy ILC networks. The analysis highlights that *FAM65B* shows highest degree of rewiring, which implies that its differential expression strongly impacts the relationships with other genes in the ILC network in response to COVID-19 infection (**Figure 2E**). Similarly, differential expression of *SELL* is also associated with significant rewiring of its interactions with a large set of genes. Interestingly, both *FAM65B* and *SELL* genes commonly change their interaction with six other genes, including *CCR2, CR2, IL12A, NINJ2, POU2AF1* and *CD40*. Among these, *CD40* alters its interaction with all three DE genes- *FAM65B*, *SELL and IFITM2.* Thus, using this differential network analysis we predict a high impact of *FAM65B* differential expression that alters its interactions with other genes, suggesting its key role in ILCs and COVID-19 infection.

**Figure 3:**
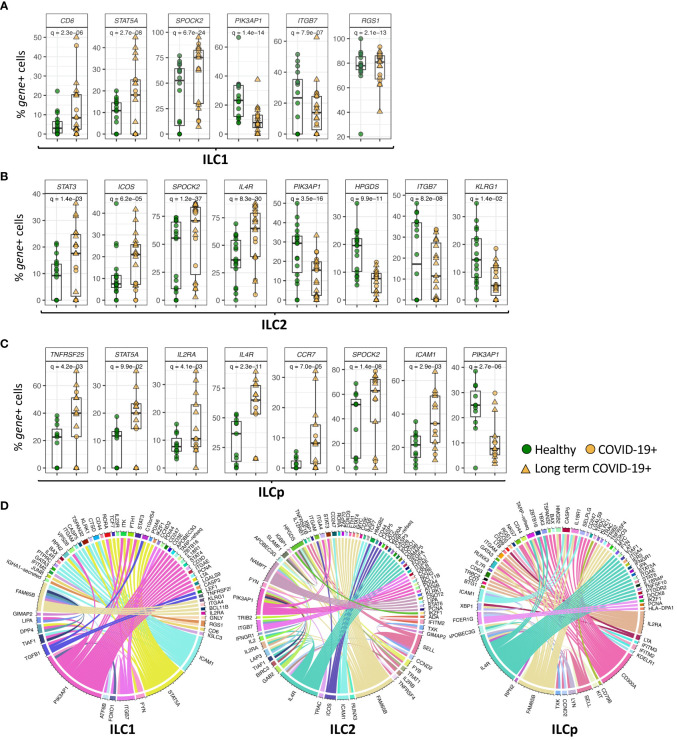
Genes differentially expressed in ILC subsets. **(A–C)** Boxplots of selected genes with statistically significant differential expression. The y-axis represents the percentage of cells expressing a given gene in each sample. The p-values were calculated by comparing the scaled gene expression profile in two groups of ILCs (COVID vs healthy control) using Seurat R package (test used: MAST) at the single cell level. **(D)** The circos plots represent differential co-expression networks reconstructed for ILC1, ILC2 and ILCp. The edges in the plot represents co-expression links significantly different across healthy vs COVID-19 ILCs. Adjusted p-values for enumerating DE genes between two groups of cells were calculated using MAST test (see methods). For boxplot representation, percentage of cells expressing the given gene in every sample is shown.

ILC1s, ILC2s and ILCps in COVID-19 patients also downregulated the gene *PIK3AP1* (adj.p= 1.4e-14, 3.5e-16 and 2.7e-06, respectively) (**Figures 2A**, **3A–C**), which encodes a signaling adapter protein linking the phosphoinositide 3-kinase (PI3K) signaling pathway to various coreceptors. In contrast to the genes downregulated in ILCs from COVID-19 patients, we observed an upregulation of *SPOCK2 in* ILC1 (adj.p = 6.7e-24), ILC2 (adj.p = 1.2e-37) and ILCp (adj. p = 1.4e-08) (**Figure 3A**). Expression of *STAT5A* (signal transducer and activator of transcription 5A), a member of the STAT family of transcription factors important in signal transduction, was upregulated in ILC1, ILC2 and ILCp cells (adj.p= 2.7e-08, 9.3e-04 and 0.09, respectively), from COVID-19 patients compared with controls (**Figures 3A, C**; **Supplementary Data File 1**).

### Genes differentially expressed in subsets of ILCs from COVID-19 patients

In addition to genes DE in all ILC subsets in COVID-19 patients, some genes were differentially expressed in specific ILC subsets. Expression of *ITGB7* (integrin subunit beta 7), which encodes an integrin superfamily member protein that plays a role in cell migration (23), was significantly downregulated in both ILC1 and ILC2 subsets (adj.p = 7.9e-07 and 8.2e-08, respectively) compared with controls (**Figures 3A, B**; **Supplementary Data File 1**). Expression of *ITGB2*, another integrin subunit encoding gene, was also downregulated in ILC1s and ILC2s (adj.p = 2.6e-10 and 1.2e-21, respectively) in COVID-19 patients compared with controls. ILC1 cells from COVID-19 patients upregulated expression of *CD6* (adj.p= 2.3e-06) (**Figure 3A**), which encodes a cell surface protein that has been shown to costimulate T cell activation and proliferation (24). ILC1s also upregulated expression of *RGS1* (adj.p= 2.1e-13), a regulator of G protein signaling (**Figure 3A**). *ICAM1* (intercellular adhesion molecule 1) was upregulated in ILC1 (p = 0.0009), ILC2 (p = 0.004) and ILCp (adj. p = 2.9e-03) subsets in COVID-19 patients (**Figure 3C**; **Supplementary Data File 1**).

ILC2 cells from COVID-19 patients downregulated the expression of *HPGDS* (adj.p = 9.9e-11), which encodes an enzyme that plays a role in the prostanoid metabolic pathway (**Figure 3B**). ILC2s from COVID-19 patients showed upregulated expression of the genes *IL4R* (adj.p =8.3e-30) and *ICOS* (adj.p = 6.2e-05), which play important roles in ILC2 homeostasis and function, as well as expression of the transcription factor *STAT3* (adj.p = 1.4e-03) (**Figure 3B**), *RORA* (adj.p = 0.001), important in development of ILC2 cells, and *NAMPT* (adj.p = 0.002) (**Supplementary Data File 1**). ILCp cells from COVID-19 patients showed upregulated expression of *IL2RA* (adj.p = 0.004), *IL4R* (adj.p = 2.3e-11) as well as other immune-related genes including *TNFRSF25* (adj.p = 4.2e-03) and *CCR7* (adj. p = 7.7e-05), which has been shown to control the migration of lymphoid cells to inflamed tissues (25) (**Figure 3C**).

Next, we attempted to identify the genes that change co-expression profiles between healthy and COVID-19 patients in each ILC subset. For each ILC subset, we constructed two co-expression networks in which each network includes the co-expression profile between every pair of genes available in our dataset. This comparative network analysis reveals the edges (i.e., co-expression links) with differential profile across healthy vs COVID-19 patients, i.e., network rewiring (**Figure 3D**). Differential co-expression analysis allowed us to predict and prioritize the genes that can alter their interactions, i.e., functional relationships, with other genes between healthy versus COVID-19 in each ILC subset. Interestingly in ILC1 cells, *ICAM1*, *PIK3AP1*, *STAT5A, TGFB1* and *FAM65B* genes show the highest degree of network rewiring across healthy vs COVID-19 correlation networks. In ILC2, we observed altered co-expression profiles of *IL4R*, *PIK3AP1*, *HPGDS*, *NAMPT*, *ICOS* and *FAM65B*. Whereas in ILCp, the differential network analysis revealed *IL2RA*, *IL4R*, *ICAM1*, *FAM65B*, *CD300A* and *FCER1G* as the most rewired genes with varying co-expression profiles across COVID-19 vs heathy co-expression networks.

### Identification of ILC specific regulons by reconstructing gene regulatory networks

**Figure 4:**
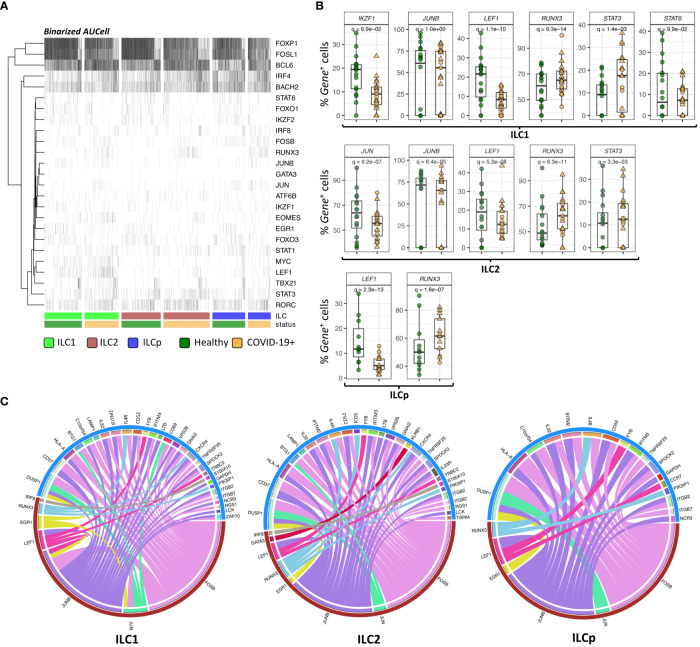
SCENIC analysis of regulon identification. **(A)** Heatmap depicting the binarized AUcell scores of 25 different TFs in ILC1, ILC2 and ILCp. The row represents TFs (regulons) and column represents ILCs. The ILCs are annotated as per the color bars in the bottom panels of the heatmap. **(B)** Boxplots of selected TF genes with statistically significant differential expression. The y-axis represents the percentage of cells expressing a given TF in each sample. The p-values were calculated by comparing the scaled gene expression profile in two groups of ILCs (COVID vs healthy control) using Seurat R package (test used: MAST) at the single cell level. **(C)** The circos plots representing reconstructed gene regulatory networks between TFs and their targets predicted in ILC1, ILC2 and ILCp cells. The red arc represents the known TFs and blue arc represents differentially expressed target genes. The edges in the plot represents regulatory interactions between TF and their target genes significantly differentially expressed across healthy vs COVID-19 ILCs. Adjusted p-values for enumerating DE genes between two groups of cells were calculated using MAST test (see methods). For boxplot representation, percentage of cells expressing the given TF gene in every sample is shown.

To investigate the potential gene expression regulators of the DE genes observed in the above analyses, we performed SCENIC analyses to predict TFs and gene target regulatory indications in the ILC subsets. From our scRNAseq panel we identified 25 regulons (TF genes) to be significantly active in most of the ILCs. For each TF gene, we calculated AUCell score in each cell that signifies the active state of the gene in a given cell (**Figure 4A**). The binarized AUcell score of 25 TFs in 6250 ILCs highlights the key TFs active in the given ILCs, especially *FOXP1*, *FOSL1*, *BCL6, IRF4* and *BACH2*. Among these, we found that 8 TFs were also DE (adj. p < 0.05) in at least one of the three ILCs, i.e., *GATA3, JKZF1, JUNB, JUN, LEF1, RUNX3, STAT3 and STAT6* (**Figure 4B**). Downregulation of *JUNB* and *LEF1*, and upregulation of *RUNX3* was observed in all three ILC subsets from COVID-19 patients. The TFs *JUN* and *STAT6* were significantly downregulated in ILC1 and ILC2, respectively. In contrast, *STAT3* is significantly upregulated in both ILC1 and ILC2.

TFs are well known to modulate the expression of their target genes without necessarily altering their own expression profile. Therefore, we reconstructed a directed gene regulatory network of TF and their target genes in ILCs using GRNboost2 and pySCENIC workflow (see methods). The network includes both DE and non-DE TFs, which can potentially regulate the expression of DE target genes. Only 7 out of 25 regulons were found to interact with DE target genes via GRNboost2 analysis (**Figure 4C**). These 7 regulons were predicted to regulate the expression profile of more than 40 genes in all the three ILCs. In ILC1, we observed 28 DE target genes strongly interacting with the 7 regulons. Of these, *FOSB, JUN and JU*N*B* were found to significantly regulate the expression profile of a large number of target genes, including *CD69* and *HLA-A*. We observed a similar trend in ILC2 and ILCp, wherein *FOSB*, *JUNB* and *RUNX3* showed the highest degree of connections with DE target genes.

## Discussion

We interrogated the transcriptional profile of CD127^+^ ILCs in peripheral blood of patients who developed asymptomatic to moderate COVID-19 following SARS-CoV-2 infection using a multi-omic approach consisting of single-cell RNA sequencing and Abseq (18). We identified 51 genes significantly differentially expressed in ILCs from COVID-19 patients compared with healthy control participants. ILC2s from COVID-19 patients had the highest number of significantly DE genes compared with control participants. The most notable DE genes included a) genes associated with anti-viral responses, b) genes that support ILC activation, proliferation, and homeostasis. Some of the genes in each of these categories were DE in ILC1s, ILC2s and ILCps, while others were DE in one or two subsets, depending on their function.

COVID-19 patients in our study consisted primarily of mild cases (*n*=17 cases), while including some asymptomatic (*n*=2) and some moderate (*n*=3) cases of COVID-19 (**Supplementary Table S1**). Previous studies which consisted of moderate and severe COVID-19 patients observed decreased frequencies of total CD127^+^ ILC (3, 13), which was especially pronounced in severe COVID-19 patients and correlated with duration of hospitalization and severity of inflammation (13, 14). Our study of ILCs from a less severe cohort allowed in-depth understanding of ILC responses to infection with SARS-CoV-2, since we did not observe decreased frequencies of total CD127^+^ ILC in our patients. We found that all ILC subsets in COVID-19 patients displayed a significantly higher frequency of CD69-expressing-cells compared with healthy controls, accompanied by a decreased frequency of cells expressing CD62L, as was previously noted for ILC2 and ILCps in COVID-19 patients (13).

Compared with healthy controls, ILCs from COVID-19 patients had DE of interferon-inducible genes with recognized anti-viral properties. We observed an upregulation of *SPOCK2*, encoding a complex proteoglycan that binds with glycosaminoglycans to form part of the extracellular matrix. SPOCK2/Testican2 production is Induced by interferon-α produced upon influenza virus infection, and has been shown to form a protective barrier against infection of neighboring cells (26). Although interferon-α produced upon infection with SARS-CoV-2 may be responsible for upregulation of *SPOCK2*, the anti-viral protective mechanism of SPOCK2 is reported to involve interaction with neuraminidase, which is absent in SARS-CoV-2 (27). *SPOCK2* is known to be expressed by T cells during anti-viral responses, but to our knowledge enhanced expression by ILCs during viral infection has not been documented previously.

Compared with healthy controls, ILC1s, ILC2s and ILCps from COVID-19 patients downregulated expression of *IFITM2* as well as *IFITM3*. Although interferon-induced transmembrane proteins (IFITMs 1, 2 and 3) have been shown to restrict infection by viral pathogens such as dengue, influenza A and Ebola virus (28), pro-viral functions of IFITM proteins have been reported for coronaviruses. SARS-CoV-2 Spike protein was shown to interact with IFITM proteins and utilize IFITM2 for efficient viral infection, while depletion of IFITM2 substantially reduced infectious virus production *in vitro* (29, 30). These observations suggest that the downregulation of *IFITM* gene expression we observed in ILCs from COVID-19 patients may be a protective mechanism.

Genes that regulate ILC activation, expansion and homeostasis were differentially expressed in ILCs from COVID-19 patients compared with healthy controls. ILC1s, ILC2s and ILCps all downregulated *FAM65B*, a gene which encodes a major target of FoxO1, a transcription factor that imposes cell quiescence. Downregulation of *FAM65B* facilitates T cell activation and proliferation (31). ILC1s, ILC2s and ILCps from COVID-19 patients upregulated expression of *STAT5A*, encoding a STAT5 protein which is activated by and mediates the responses of many cytokines and growth hormones (32). ILC2s upregulated expression of *ICOS*, which plays a critical role in ILC2 homeostasis and function (33). ICOS: ICOS-Ligand interaction promotes survival of ILC2s and type 2 cytokine production through signaling mediated by STAT5. ILC2s and ILCps from COVID-19 patients, which contain precursors of ILC2s, upregulated the *IL4R*, which by binding IL-4, promotes ILC2 proliferation and enhances production of IL-4 and IL13 (21, 34). ILC2s upregulated *RORA*, encoding the transcription factor RAR-related orphan nuclear receptor alpha, which is required for development of ILC2s (35). ILCps upregulated expression of *IL2RA*, the gene encoding the IL-2 receptor alpha chain, which by binding IL-2, could promote expansion of ILCps and potentiate the effects of other cytokines on ILCps. ILC1s upregulated expression of *RGS1*, encoding a member of the regulator of G-protein signaling family that attenuates the signaling activity of G-proteins (36). ILC1s upregulated expression of *CD6*, encoding the cell surface protein CD6, which by interaction with the adhesion molecule CD166 stimulates and supports T cell activation (24, 37), although negative influences of CD6 on T cell activation have also been reported (24). ILC1s upregulated *TGFB1*, which encodes a secreted ligand of the TGFβ family with potent anti-inflammatory functions. TGF-β, a pleiotropic cytokine which is often upregulated in infection or inflammatory conditions, regulates cell proliferation, differentiation and homeostasis of effector and regulatory T cells as well as other immune cells (38).

Expression of *ICAM1*, encoding an adhesion molecule that is the major human rhinovirus receptor, was upregulated in all ILC subsets from COVID patients compared with controls. ICAM-1 is a cell surface glycoprotein upregulated by endothelial, epithelial and immune cells in response to inflammation (39), which when expressed by T cells can deliver a costimulatory signal leading to T cell activation (40). ICAM-1 expressed by activated ILC2s has been shown to have a significant effect on ILC2 activation, proliferation and cytokine secretion, but is not required for ILC2 migration to the lung (41).

Other genes DE in COVID-19 patients compared with controls were involved in lymphocyte migration. ILC1 and ILC2 subsets downregulated expression of *ITGB7* (integrin subunit beta 7), which encodes the β chain of a member of the integrin superfamily of adhesion receptors that play a role in leukocyte adhesion and function in cell signaling (42). ILC2s also downregulated expression of *ITGB2*, another integrin subunit coding gene. ILCps in COVID-19 patients upregulated *CCR7* gene expression compared with controls. *CCR7* encodes a receptor which is a key regulator of lymphoid cell migration to inflamed tissues and secondary lymphoid organs (25, 43). Interestingly, CCR7 was initially identified as a gene induced on lymphoid cells by Epstein-Barr virus (EBV) infection.

Differential expression of transcription factors in COVID patients compared with healthy controls was also observed. As noted above, *STAT5A* was upregulated in all ILC subsets. The *STAT3* gene encoding transcription factor STAT3 was upregulated in ILC1s and ILC2s from COVID-19 patients. Studies suggest that hyperactivated STAT3 is key to pathology observed in COVID-19 patients, since STAT3 acts in a positive feedback loop with plasminogen activator inhibitor (PAI-1) leading to coagulopathy characterized by intravascular thrombi (44 **).**

In summary, this study provides an in-depth probe of genes DE in ILCs from COVID-19 patients compared with control participants. ILCs were not depleted in our patient samples, which were obtained primarily from patients with mild disease. The DE analysis showed that ILCs from COVID-19 patients differed transcriptionally from those of healthy control patients. Interestingly, although some genes were DE uniquely by one or two ILC subsets, many of the most significant DE genes observed were DE in common by all the subsets examined (ILC1, ILC2 and ILCp). These included *IFITM2 and IFITM3, IFIT* family members which play a role in viral infection and *SPOCK2*, upregulated upon virus infection and recognized as protective against influenza infection. Other genes significantly DE by COVID-19 patients were genes that favored ILC homeostasis, activation, and expansion. Taken together, the genes DE in COVID-19 compared with healthy participants suggest that ILCs in PB of COVID-19 patients are activated, expanding and mobilized in response to SARS-CoV-2 infection. The findings were consistent when comparing ILCs from healthy participants with those from long-term COVID-19 participants. Additionally, we observed shared biological processes and commonly coordinated dysregulation across all three ILC subtypes, indicating a concerted effort to mount an immune response against SARS-CoV-2.

This study also had some limitations. The number of genes analyzed in the multimodal single cell panel was limited to 430, so the differentially expressed genes and enriched pathways may not fully represent all affected biological processes in each ILC subset. Additionally, with only 430 genes, differentially expressed transcriptional targets of predicted transcription factors could not be identified comprehensively.

## Materials and methods

### Study participants, blood draws and processing

Participants were recruited as described previously (45) from adults who had a positive SARS-COV-2 RT-PCR test at Stanford Health Care (NCT04373148). Collection of Covid samples occurred between May to December 2020. The cohort used in this study consisted of asymptomatic (n=2), mild (n=17), and moderate (n=3) COVID-19 infections, some of whom developed long term COVID-19 (n=15). The clinical case severities at the time of diagnosis were defined as asymptomatic, moderate or mild according to the guidelines released by NIH (46). Long term (LT) COVID was defined as symptoms occurring 30 or more days after infection, consistent with CDC guidelines (47, 48). Some participants in our study continued to have LT COVID symptoms 90 days after diagnosis (n=12). Exclusion criteria for COVID sample study were NIH severity diagnosis of severe or critical at the time of positive covid test. Samples selected for this study were obtained within 76 days of positive PCR COVID-19 test date. Healthy controls were selected who had sample collection before 2020. Informed consent was obtained from all participants. All protocols were approved by the Stanford Administrative Panel on Human Subjects in Medical Research. Peripheral blood was drawn by venipuncture and using validated and published procedures (49), peripheral blood mononuclear cells (PBMCs) were isolated by Ficoll-based density gradient centrifugation, frozen in aliquots and stored in liquid nitrogen at -80°C, until thawing. A summary of participant demographics is presented in **Supplementary Table S1**.

### ILC Enrichment, single cell captures for Abseq and targeted mRNAseq

PBMC samples from 22 COVID-19+ patients and 25 healthy individuals were thawed, and each sample stained with Sample Tag (BD #633781) at room temperature for 20 minutes. Samples were combined in healthy control or COVID-19 tubes. Cells were surface stained with a panel of fluorochrome-conjugated antibodies (**Supplementary Table S2**) in buffer (PBS with 0.25% BSA and 1mM EDTA) for 20 minutes at room temperature prior to immunomagnetic negative selection for ILCs. Following ILC enrichment using the EasySep human Pan-ILC enrichment kit (StemCell Technologies #17975), cells from healthy and COVID-19 recovered participants were counted and normalized before combining. ILCs were sorted using a BD FACS Aria at the Stanford FACS facility prior to incubation with AbSeq oligo-linked mAbs (**Supplementary Figure 1A**; **Supplementary Table S4**). The flow gating for sorting ILCs was kept lenient (bigger gates were used) as the number of ILCs are smaller in peripheral blood (1–2%), this was done to ensure so that we capture most of ILCs, even if gating extend to capture other cell population (e.g. B cells). These contaminations were later removed using the Abseq gating (see below) to keep specific ILC population. Sorted cells were processed by the Stanford Human Immune Monitoring Center (HIMC) using the BD Rhapsody platform (19). Library was prepared using the BD Immune Response Targeting Panel (BD Kit #633750) with addition of custom gene panel reagents (**Supplementary Table S5**) and sequenced on Illumina NovaSeq 6000 at Stanford Genomics Sequencing Center (SGSC). ILCs were identified as Lineage^neg^ (CD3^neg^, CD14^neg^, CD34^neg^, CD19^neg^), NKG2A^neg^, CD45^+^ and ILCs further defined as CD127^+^CD161^+^ and as subsets: ILC1 (CD117^neg^CRTH2^neg^), ILC2 (CRTH2^+^) and ILCp (CD117^+^CRTH2^neg^) (**Supplementary Figure 1B**).

### Computational data analysis

The above multi-modal setup allowed paired measurements of cellular transcriptome and cell surface protein abundance. The ILC1, ILC2 and ILCp cells were manually gated based on the abundance profile of CD127, CD117, CD161 and CRTH2 (**Supplementary Figure 1B**). Before the integrative analysis, the complete multi-modal single cell dataset containing ILC subsets was converted into single Seurat object. All the subsequent protein-level and gene-level analyses were performed using multimodal data analysis pipeline of Seurat R package version 4.0 (50). The normalized and scaled protein abundance profile was used for estimating the integrated harmony dimensions using *runHarmony* function in Seurat R package (reduction= ‘apca’ and group.by.vars = ‘batch’). The batch corrected harmony embeddings were then used for computing the Uniform Manifold Approximation and Projection (UMAP) dimensions to visualize the clusters of ILC subsets. Differential marker analysis of surface proteins, between two groups of cells (COVID-19 and Healthy cohort), from abseq panels was computed with normalized and scaled expression values using *FindMarkers* function from Seurat R package (test.use=‘wilcox’). Similarly, differential gene expression was performed on normalized and scaled gene expression values from between two groups of cells (COVID-19 and Healthy cohort) using the *FindMarkers* function from Seurat R package (test.use=‘MAST’ and latent.vars=‘batch’). Genes with log-fold change > 0.5 and adjusted p-value < 0.05 (method: Benjamini-Hochberg) (51) were considered as significant for further evaluation. The resulting adjusted p-values box-plots were plotted using ggplot2 R package (version 3.4.2) (52) after computing the number of cells expressing a given protein or gene in each sample. Pathway enrichment analysis of DE genes was performed using web-server metascape (version 3.5) (53). The AUCells score and gene regulatory network analysis was performed using pySCENIC pipeline (version 0.12.1) (54). Gene regulatory network was reconstructed using GRNBoost2 algorithm (55) and the list of TFs in humans (genome version: hg38) were obtained from cisTarget database. (https://resources.aertslab.org/cistarget) (56). Cellular enrichment (aka AUCell) analysis that measures the activity of TF or gene signatures across all single cells was performed using aucell function in pySCENIC python library. The ggplot2 R package (version 3.4.2) was used for boxplot visualization. The differential gene co-expression analysis was performed using scSFMnet R package which uses single cell gene count matrix as input and reconstruct condition specific (COVID-19+ and healthy) gene interaction network (57). The approach not only allowed us to build co-expression networks using discrete single cell dataset, but also allowed us to compare two networks using (default parameters). The outcome of this analysis was a differential co-expression network containing only the gene-gene interactions that significantly differed between ILCs of COVID-19+ versus healthy participants. Here, four differential network were reconstructed using the gene expression profile of either total ILCs or ILC1 or ILC2 or ILCp. Circular plots were generated using the R package circlize (version 0.4.15) (58).

## Data availability statement

The data presented in the study are deposited in the DRYAD data repository, DOI https://doi.org/10.5061/dryad.8931zcrz4.

## Ethics statement

The studies involving humans were approved by Stanford Administrative Panel on Human Subjects in Medical Research. The studies were conducted in accordance with the local legislation and institutional requirements. The participants provided their written informed consent to participate in this study.

## Author contributions

AK: Conceptualization, Data curation, Formal analysis, Methodology, Validation, Visualization, Writing – original draft, Writing – review & editing. IC: Conceptualization, Investigation, Methodology, Writing – review & editing. XH: Methodology, Validation, Writing – review & editing. ZH: Data curation, Formal analysis, Investigation, Methodology, Writing – review & editing. ZK: Investigation, Methodology, Resources, Supervision, Writing – review & editing. XJ: Methodology, Software, Writing – review & editing. SC: Formal analysis, Methodology, Writing – review & editing. CA: Conceptualization, Investigation, Project administration, Resources, Supervision, Writing – review & editing. SB: Conceptualization, Investigation, Project administration, Resources, Supervision, Writing – review & editing. BP: Investigation, Resources, Supervision, Writing – review & editing. HM: Resources, Writing – review & editing. MD: Conceptualization, Writing – review & editing. RC: Conceptualization, Investigation, Project administration, Resources, Supervision, Writing – review & editing. RD: Conceptualization, Formal analysis, Investigation, Methodology, Supervision, Visualization, Writing – original draft, Writing – review & editing. KN: Conceptualization, Funding acquisition, Investigation, Methodology, Project administration, Resources, Supervision, Validation, Writing – original draft, Writing – review & editing.

## References

[1] Huang C Wang Y Li X Ren L Zhao J Hu Y. Clinical features of patients infected with 2019 novel coronavirus in Wuhan, China. Lancet. (2020) 395:497–506. doi: 10.1016/S0140-6736(20)30183-5 31986264 PMC7159299

[2] Merad M Blish CA Sallusto F Iwasaki A. The immunology and immunopathology of COVID-19. Science. (2022) 375:1122–7. doi: 10.1126/science.abm8108 PMC12828912 35271343

[3] Silverstein NJ Wang Y Manickas-Hill Z Carbone C Dauphin A Boribong BP. Innate lymphoid cells and COVID-19 severity in SARS-CoV-2 infection. Elife. (2022) 11. doi: 10.7554/eLife.74681 PMC9038195 35275061

[4] Arunachalam PS Wimmers F Mok CKP Perera R Scott M Hagan T. Systems biological assessment of immunity to mild versus severe COVID-19 infection in humans. Science. (2020) 369:1210–20. doi: 10.1126/science.abc6261 PMC7665312 32788292

[5] Azkur AK Akdis M Azkur D Sokolowska M van de Veen W Brüggen MC. Immune response to SARS-CoV-2 and mechanisms of immunopathological changes in COVID-19. Allergy. (2020) 75:1564–81. doi: 10.1111/all.14364 PMC7272948 32396996

[6] Qin C Zhou L Hu Z Zhang S Yang S Tao Y. Dysregulation of immune response in patients with coronavirus 2019 (COVID-19) in Wuhan, China. Clin Infect Dis. (2020) 71:762–8. doi: 10.1093/cid/ciaa248 PMC7108125 32161940

[7] Wilk AJ Rustagi A Zhao NQ Roque J Martínez-Colón GJ McKechnie JL. A single-cell atlas of the peripheral immune response in patients with severe COVID-19. Nat Med. (2020) 26:1070–6. doi: 10.1038/s41591-020-0944-y PMC7382903 32514174

[8] Zheng HY Zhang M Yang CX Zhang N Wang XC Yang XP. Elevated exhaustion levels and reduced functional diversity of T cells in peripheral blood may predict severe progression in COVID-19 patients. Cell Mol Immunol. (2020) 17:541–3. doi: 10.1038/s41423-020-0401-3 PMC7091621 32203186

[9] Lim AI Li Y Lopez-Lastra S Stadhouders R Paul F Casrouge A. Systemic human ILC precursors provide a substrate for tissue ILC differentiation. Cell. (2017) 168:1086–1100.e1010. doi: 10.1016/j.cell.2017.02.021 28283063

[10] Vivier E Artis D Colonna M Diefenbach A Di Santo JP Eberl G. Innate lymphoid cells: 10 years on. Cell. (2018) 174:1054–66. doi: 10.1016/j.cell.2018.07.017 30142344

[11] Klose CSN Artis D. Innate lymphoid cells control signaling circuits to regulate tissue-specific immunity. Cell Res. (2020) 30:475–91. doi: 10.1038/s41422-020-0323-8 PMC7264134 32376911

[12] Nagasawa M Heesters BA Kradolfer CMA Krabbendam L Martinez-Gonzalez I de Bruijn MJW. KLRG1 and NKp46 discriminate subpopulations of human CD117(+)CRTH2(-) ILCs biased toward ILC2 or ILC3. J Exp Med. (2019) 216:1762–76. doi: 10.1084/jem.20190490 PMC6683990 31201208

[13] García M Kokkinou E Carrasco García A Parrot T Palma Medina LM Maleki KT. Innate lymphoid cell composition associates with COVID-19 disease severity. Clin Transl Immunol. (2020) 9:e1224. doi: 10.1002/cti2.1224 PMC7734472 33343897

[14] Kuri-Cervantes L Pampena MB Meng W Rosenfeld AM Ittner CAG Weisman AR. Comprehensive mapping of immune perturbations associated with severe COVID-19. Sci Immunol. (2020) 5:eabd7114. doi: 10.1126/sciimmunol.abd7114 32669287 PMC7402634

[15] Chang YJ Kim HY Albacker LA Baumgarth N McKenzie AN Smith DE. Innate lymphoid cells mediate influenza-induced airway hyper-reactivity independently of adaptive immunity. Nat Immunol. (2011) 12:631–8. doi: 10.1038/ni.2045 PMC3417123 21623379

[16] Monticelli LA Sonnenberg GF Abt MC Alenghat T Ziegler CG Doering TA. Innate lymphoid cells promote lung-tissue homeostasis after infection with influenza virus. Nat Immunol. (2011) 12:1045–54. doi: 10.1038/ni.2131 PMC3320042 21946417

[17] Wills-Karp M Finkelman FD. Innate lymphoid cells wield a double-edged sword. Nat Immunol. (2011) 12:1025–7. doi: 10.1038/ni.2142 22012433

[18] Shahi P Kim SC Haliburton JR Gartner ZJ Abate AR. Abseq: Ultrahigh-throughput single cell protein profiling with droplet microfluidic barcoding. Sci Rep. (2017) 7:44447. doi: 10.1038/srep44447 28290550 PMC5349531

[19] Mair F Erickson JR Voillet V Simoni Y Bi T Tyznik AJ. A targeted multi-omic analysis approach measures protein expression and low-abundance transcripts on the single-cell level. Cell Rep. (2020) 31:107499. doi: 10.1016/j.celrep.2020.03.063 32268080 PMC7224638

[20] Björklund Å.K Forkel M Picelli S Konya V Theorell J Friberg D. The heterogeneity of human CD127(+) innate lymphoid cells revealed by single-cell RNA sequencing. Nat Immunol. (2016) 17:451–60. doi: 10.1038/ni.3368 26878113

[21] Bal SM Bernink JH Nagasawa M Groot J Shikhagaie MM Golebski K. IL-1β, IL-4 and IL-12 control the fate of group 2 innate lymphoid cells in human airway inflammation in the lungs. Nat Immunol. (2016) 17:636–45. doi: 10.1038/ni.3444 27111145

[22] Yang S Liu F Wang QJ Rosenberg SA Morgan RA. The shedding of CD62L (L-selectin) regulates the acquisition of lytic activity in human tumor reactive T lymphocytes. PLoS One. (2011) 6:e22560. doi: 10.1371/journal.pone.0022560 21829468 PMC3145643

[23] Yu Y Zhu J Mi LZ Walz T Sun H Chen J. Structural specializations of α(4)β(7), an integrin that mediates rolling adhesion. J Cell Biol. (2012) 196:131–46. doi: 10.1083/jcb.201110023 PMC3255974 22232704

[24] Mori D Grégoire C Voisinne G Celis-Gutierrez J Aussel R Girard L. The T cell CD6 receptor operates a multitask signalosome with opposite functions in T cell activation. J Exp Med. (2021) 218:e20201011. doi: 10.1084/jem.20201011 33125054 PMC7608068

[25] Förster R Davalos-Misslitz AC Rot A. CCR7 and its ligands: balancing immunity and tolerance. Nat Rev Immunol. (2008) 8:362–71. doi: 10.1038/nri2297 18379575

[26] Ahn N Kim WJ Kim N Park HW Lee SW Yoo JY. The interferon-inducible proteoglycan testican-2/SPOCK2 functions as a protective barrier against virus infection of lung epithelial cells. J Virol. (2019) 93:e00662-19. doi: 10.1128/JVI.00662-19 31341044 PMC6798107

[27] Robson B. Bioinformatics studies on a function of the SARS-CoV-2 spike glycoprotein as the binding of host sialic acid glycans. Comput Biol Med. (2020) 122:103849. doi: 10.1016/j.compbiomed.2020.103849 32658736 PMC7278709

[28] Bailey CC Zhong G Huang IC Farzan M. IFITM-family proteins: The cell's first line of antiviral defense. Annu Rev Virol. (2014) 1:261–83. doi: 10.1146/annurev-virology-031413-085537 PMC4295558 25599080

[29] Nchioua R Schundner A Kmiec D Prelli Bozzo C Zech F Koepke L. SARS-CoV-2 variants of concern hijack IFITM2 for efficient replication in human lung cells. J Virol. (2022) 96:e0059422. doi: 10.1128/jvi.00594-22 35543509 PMC9175628

[30] Prelli Bozzo C Nchioua R Volcic M Koepke L Krüger J Schütz D. IFITM proteins promote SARS-CoV-2 infection and are targets for virus inhibition. vitro. Nat Commun. (2021) 12:4584. doi: 10.1038/s41467-021-24817-y 34321474 PMC8319209

[31] Froehlich J Versapuech M Megrelis L Largeteau Q Meunier S Tanchot C. FAM65B controls the proliferation of transformed and primary T cells. Oncotarget. (2016) 7:63215–25. doi: 10.18632/oncotarget.v7i39 PMC5325358 27556504

[32] Lin JX Leonard WJ. The role of Stat5a and Stat5b in signaling by IL-2 family cytokines. Oncogene. (2000) 19:2566–76. doi: 10.1038/sj.onc.1203523 10851055

[33] Maazi H Patel N Sankaranarayanan I Suzuki Y Rigas D Soroosh P. ICOS : ICOS-ligand interaction is required for type 2 innate lymphoid cell function, homeostasis, and induction of airway hyperreactivity. Immunity. (2015) 42:538–51. doi: 10.1016/j.immuni.2015.02.007 PMC4366271 25769613

[34] Baba R Kabata H Shirasaki Y Kamatani T Yamagishi M Irie M. Upregulation of IL-4 receptor signaling pathway in circulating ILC2s from asthma patients. J Allergy Clin Immunology: Global. (2022) 1:299–304. doi: 10.1016/j.jacig.2022.07.007 PMC10509846 37779537

[35] Halim TY MacLaren A Romanish MT Gold MJ McNagny KM Takei F. Retinoic-acid-receptor-related orphan nuclear receptor alpha is required for natural helper cell development and allergic inflammation. Immunity. (2012) 37:463–74. doi: 10.1016/j.immuni.2012.06.012 22981535

[36] Moratz C Kang VH Druey KM Shi CS Scheschonka A Murphy PM. Regulator of G protein signaling 1 (RGS1) markedly impairs Gi alpha signaling responses of B lymphocytes. J Immunol. (2000) 164:1829–38. doi: 10.4049/jimmunol.164.4.1829 10657631

[37] Hassan NJ Simmonds SJ Clarkson NG Hanrahan S Puklavec MJ Bomb M. CD6 regulates T-cell responses through activation-dependent recruitment of the positive regulator SLP-76. Mol Cell Biol. (2006) 26:6727–38. doi: 10.1128/MCB.00688-06 PMC1592849 16914752

[38] Li MO Flavell RA. Contextual regulation of inflammation: a duet by transforming growth factor-beta and interleukin-10. Immunity. (2008) 28:468–76. doi: 10.1016/j.immuni.2008.03.003 18400189

[39] Bui TM Wiesolek HL Sumagin R. ICAM-1: A master regulator of cellular responses in inflammation, injury resolution, and tumorigenesis. J Leukoc Biol. (2020) 108:787–99. doi: 10.1002/JLB.2MR0220-549R PMC7977775 32182390

[40] Chirathaworn C Kohlmeier JE Tibbetts SA Rumsey LM Chan MA Benedict SH. Stimulation through intercellular adhesion molecule-1 provides a second signal for T cell activation. J Immunol. (2002) 168:5530–7. doi: 10.4049/jimmunol.168.11.5530 12023348

[41] Hurrell BP Howard E Galle-Treger L Helou DG Shafiei-Jahani P Painter JD. Distinct roles of LFA-1 and ICAM-1 on ILC2s control lung infiltration, effector functions, and development of airway hyperreactivity. Front Immunol. (2020) 11:542818. doi: 10.3389/fimmu.2020.542818 33193309 PMC7662114

[42] Tyler CJ Guzman M Lundborg LR Yeasmin S Zgajnar N Jedlicka P. Antibody secreting cells are critically dependent on integrin α4β7/MAdCAM-1 for intestinal recruitment and control of the microbiota during chronic colitis. Mucosal Immunol. (2022) 15:109–19. doi: 10.1038/s41385-021-00445-z PMC8732264 34433904

[43] Yan Y Chen R Wang X Hu K Huang L Lu M. CCL19 and CCR7 expression, signaling pathways, and adjuvant functions in viral infection and prevention. Front Cell Dev Biol. (2019) 7:212. doi: 10.3389/fcell.2019.00212 31632965 PMC6781769

[44] Matsuyama T Kubli SP Yoshinaga SK Pfeffer K Mak TW. An aberrant STAT pathway is central to COVID-19. Cell Death Differ. (2020) 27:3209–25. doi: 10.1038/s41418-020-00633-7 PMC7545020 33037393

[45] Jia X Cao S Lee AS Manohar M Sindher SB Ahuja N. Anti-nucleocapsid antibody levels and pulmonary comorbid conditions are linked to post-COVID-19 syndrome. JCI Insight. (2022) 7:e156713. doi: 10.1172/jci.insight.156713 35801588 PMC9310538

[46] NIH. Clinical Spectrum of SARS-CoV-2 Infection. National Institute of Health (2023). Bethesda, Maryland. Available at: https://www.covid19treatmentguidelines.nih.gov/overview/clinical-spectrum/.

[47] CDC. Long COVID or Post-COVID Conditions. Centers for Disease Control and Prevention (2023). Available at: https://www.cdc.gov/coronavirus/2019-ncov/long-term-effects/.

[48] COVID.gov. About Long COVID: Terms & Definitions (2023). Available online at: https://www.covid.gov/be-informed/longcovid/about.

[49] Fuss IJ Kanof ME Smith PD Zola H. Isolation of whole mononuclear cells from peripheral blood and cord blood. Curr Protoc Immunol. (2009) 7:7.1.1–7.1.8. doi: 10.1002/0471142735.im0701s85 19347849

[50] Hao Y Hao S Andersen-Nissen E Mauck WM 3rd Zheng S Butler A. Integrated analysis of multimodal single-cell data. Cell. (2021) 184:3573–3587.e3529. doi: 10.1016/j.cell.2021.04.048 34062119 PMC8238499

[51] Benjamini Y Hochberg Y. Controlling the false discovery rate: a practical and powerful approach to multiple testing. J R Stat society: Ser B (Methodological). (1995) 57:289–300. doi: 10.1111/j.2517-6161.1995.tb02031.x

[52] Wickham H. ggplot2: Elegant Graphics for Data Analysis. New York: Springer-Verlag (2016). doi: 10.1007/978-3-319-24277-4

[53] Zhou Y Zhou B Pache L Chang M Khodabakhshi AH Tanaseichuk O. Metascape provides a biologist-oriented resource for the analysis of systems-level datasets. Nat Commun. (2019) 10:1523. doi: 10.1038/s41467-019-09234-6 30944313 PMC6447622

[54] Aibar S González-Blas CB Moerman T Huynh-Thu VA Imrichova H Hulselmans G. SCENIC: single-cell regulatory network inference and clustering. Nat Methods. (2017) 14:1083–6. doi: 10.1038/nmeth.4463 PMC5937676 28991892

[55] Moerman T Aibar Santos S Bravo González-Blas C Simm J Moreau Y Aerts J. GRNBoost2 and Arboreto: efficient and scalable inference of gene regulatory networks. Bioinformatics. (2019) 35:2159–61. doi: 10.1093/bioinformatics/bty916 30445495

[56] Herrmann C Van de Sande B Potier D Aerts S. i-cisTarget: an integrative genomics method for the prediction of regulatory features and cis-regulatory modules. Nucleic Acids Res. (2012) 40:e114. doi: 10.1093/nar/gks543 22718975 PMC3424583

[57] Sekula M Gaskins J Datta S. Single-cell differential network analysis with sparse bayesian factor models. Front Genet. (2021) 12:810816. doi: 10.3389/fgene.2021.810816 35186014 PMC8855158

[58] Gu Z Gu L Eils R Schlesner M Brors B. circlize Implements and enhances circular visualization in R. Bioinformatics. (2014) 30:2811–2. doi: 10.1093/bioinformatics/btu393 24930139

