# Supplementary material for: Single cell multi-omic analysis identifies key genes differentially expressed in innate lymphoid cells from COVID-19 patients
Source: Front Immunol. 2024 Jul 4;15:1374828. doi: 10.3389/fimmu.2024.1374828 (PMC11255397; doi:10.3389/fimmu.2024.1374828)
Supplement: Supplementary file 1 [file DataSheet_1.pdf]

a.

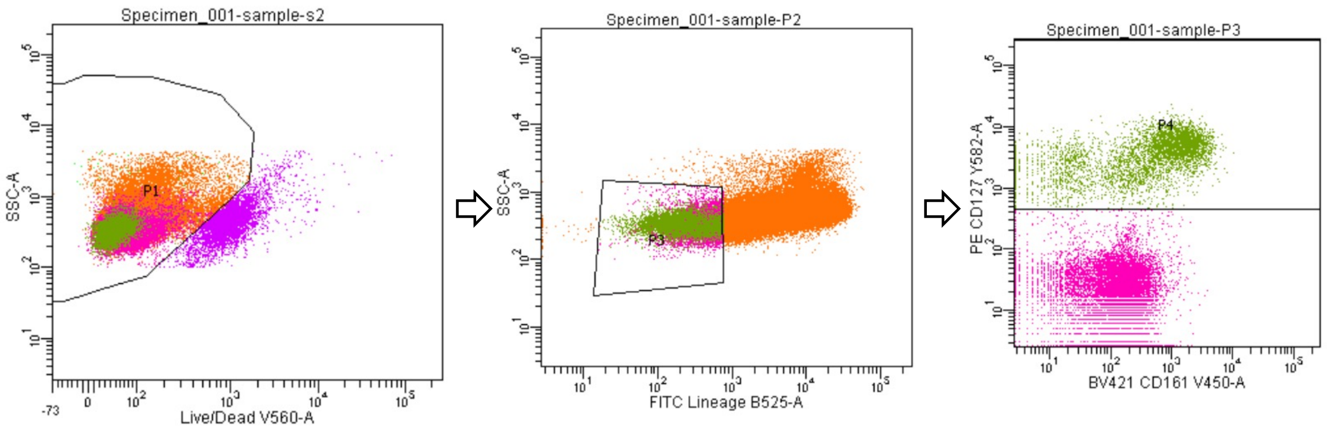

b.

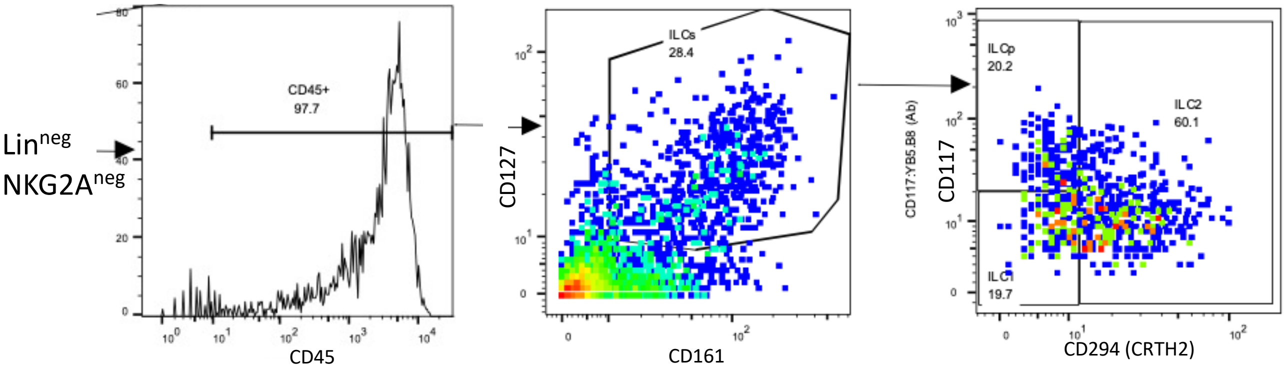

Supplementary Fig. 1 A. FACS Cell sorting schema to identify ILCs prior to Abseq & scRNAseq profiling using BD Rhapsody multi-omics platform. **B. Gating strategy for ILC identification**. Using Abseq oligonucleotide-linked antibodies, ILCs were identified as Lineage^{neg}, NKG2A^{neg} and CD45^{+}. ILCs were further defined as CD127^{+}CD161^{+} as well as ILC subsets: ILC1 (CD117^{neg}CRTH2^{neg}), ILC2 (CRTH2^{+}) and ILCp (CD117^{+}CRTH2^{neg}).

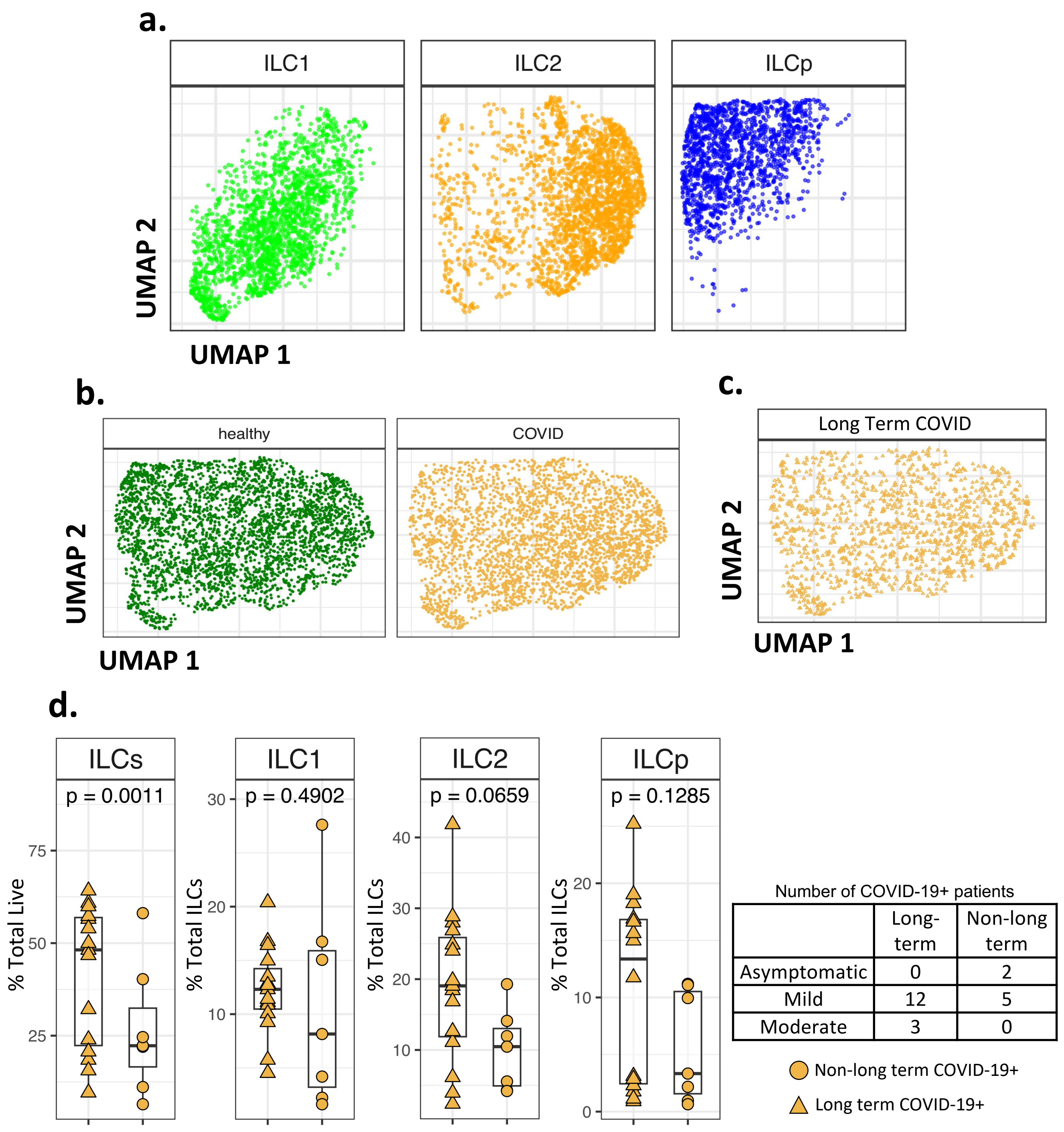

Supplementary Fig. 2. (a) UMAP directional reduction analysis of manually gated ILCs from COVID-19 patients and healthy control participants. The ILC1, ILC2 and ILCp cells are represented separately to show their distribution across the entire UMAP as shown in Fig 1b. (**b**) UMAP analysis of ILCs from healthy compared with COVID-19 patients. The ILCs are uniformly distributed in both groups of participants. For UMAP analysis 6247 single cells were plotted of which 2103 cells were ILC1 , 2509 were ILC2 and 1635 were ILCp. **(c)** UMAP analysis of ILCs from patients with long-term COVID-19 symptoms. The ILCs are uniformly distributed across the entire UMAP. (**d**) Frequencies (i.e., percentage) of ILCs and ILC subsets from 22 COVID-19 patients were compared with patients with long-term COVID-19 symptoms (*n*=15) versus non-long-term covid (*n*=7). P-values between two groups of samples were calculated using Wilcoxon rank-sum test.

Supplementary Fig. 3

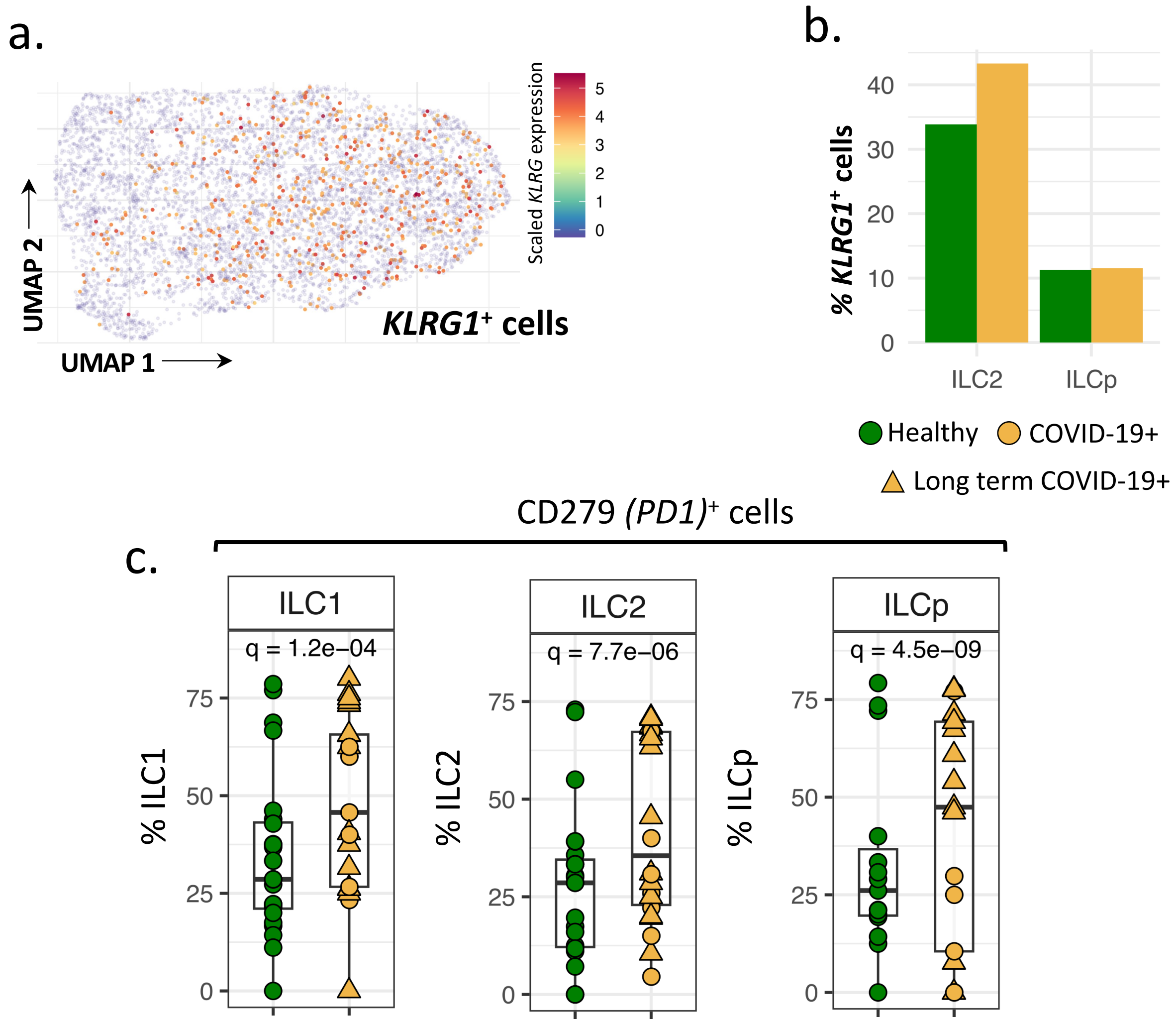

Supplementary Fig. 3. (a) UMAP analysis shows the expression profile of *KLRG* in ILCs*.* (**b**) The number of *KLRG1*+ ILC2 and ILCp cells across healthy control vs COVID-19 patients. (**c**) Differences in frequency of CD279 (*PD1*)+ cells by ILC subsets from COVID-19 patients compared with healthy controls. For boxplot representation, percentage of cells expressing CD279 protein in every sample is shown..

Supplementary Fig. 4

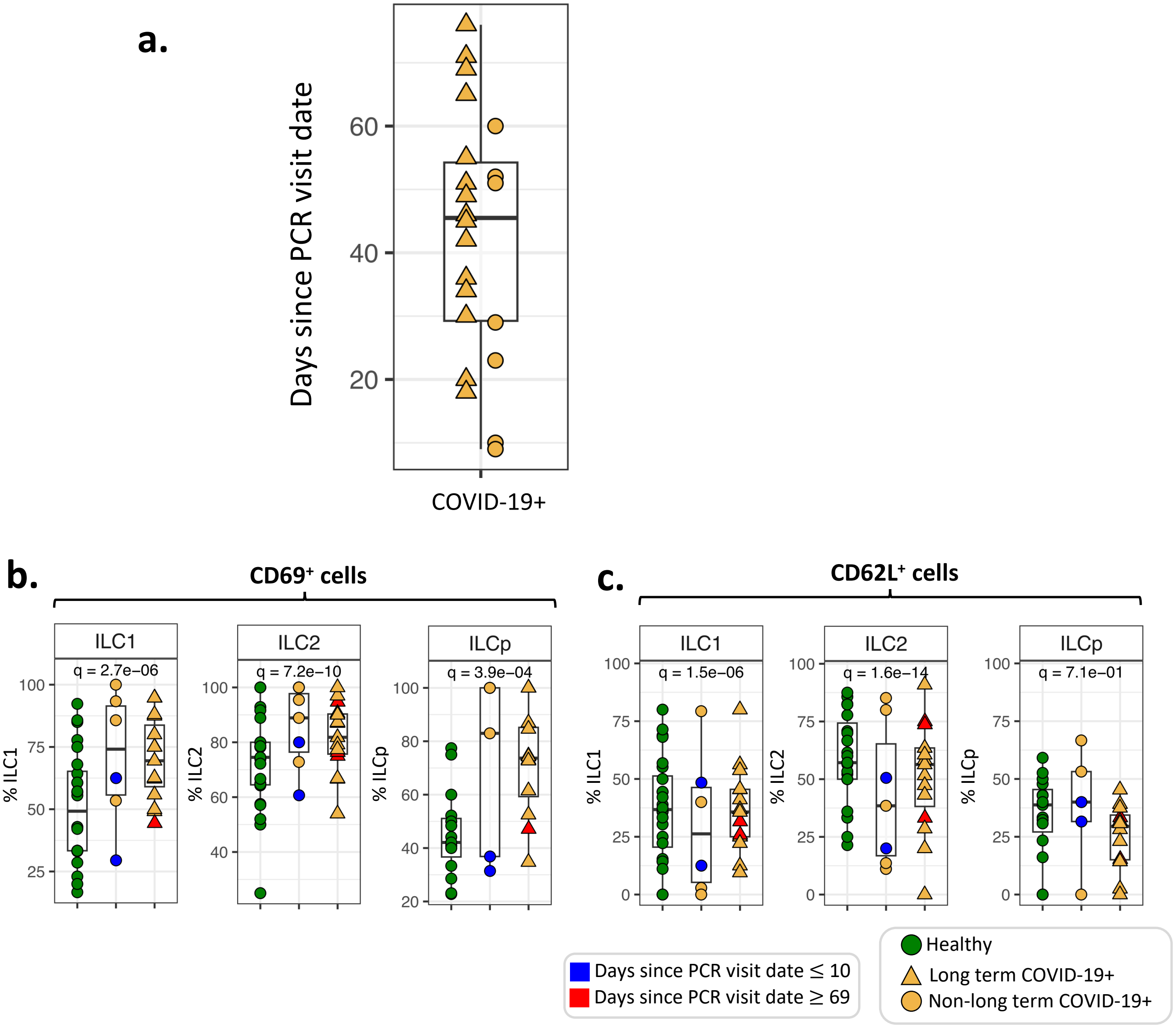

Supplementary Fig. 4. Days since PCR visit date. (a) The box plot shows the number of days post COVID-19 RT-PCR positive diagnosis at which patient samples were taken . **(b-c)** Differences in frequency of CD69^{+} cells and CD62L (*SELL*)$^{+}$ cells by ILC subsets from COVID-19 patients compared with healthy controls (as also represented in Fig. 1e & g). Samples from COVID-19 patients taken less than 11 days after their COVID-19 RT-PCR diagnosis are colored in blue, whereas samples taken from COVID-19 patients $\geq$ 68 days post RT-PCR diagnosis are colored in red. Adjusted p-values between two groups of cells were calculated using Wilcoxon rank-sum test (see methods). For boxplot representation, percentage of cells expressing a given protein in every sample is shown.

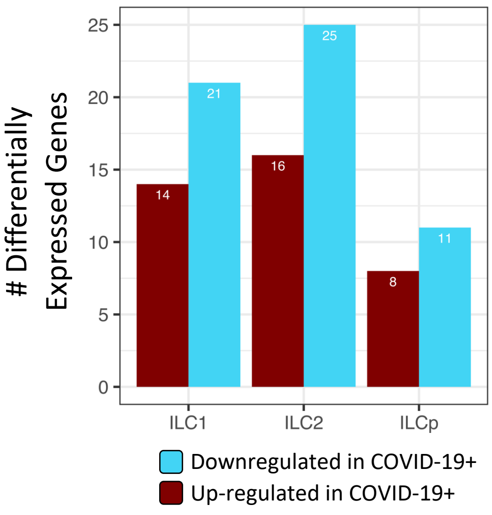

Supplementary Fig. 5. Number of up-regulated and down-regulated genes in each ILC subset.

Supplementary Fig. 6

a. **ILC1: Enrichment Analysis of differentially expressed genes (*n=32*)**

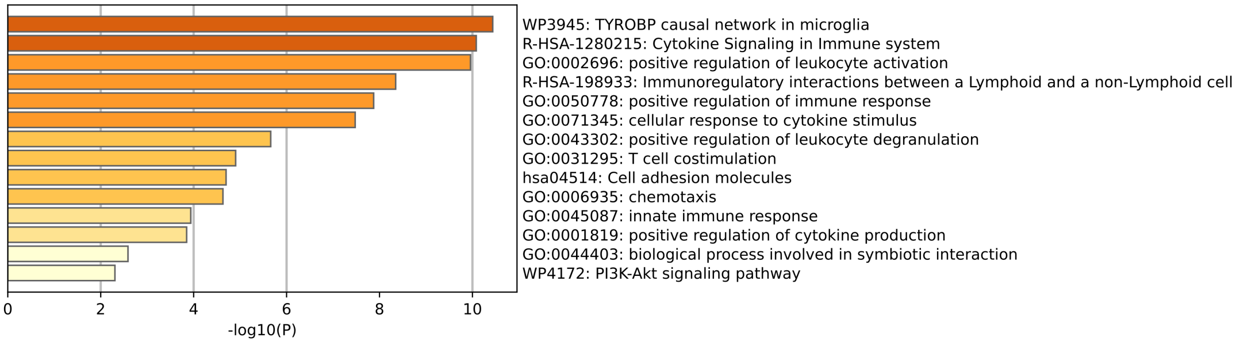

b. **ILC2: Enrichment Analysis of differentially expressed genes (*n=38*)**

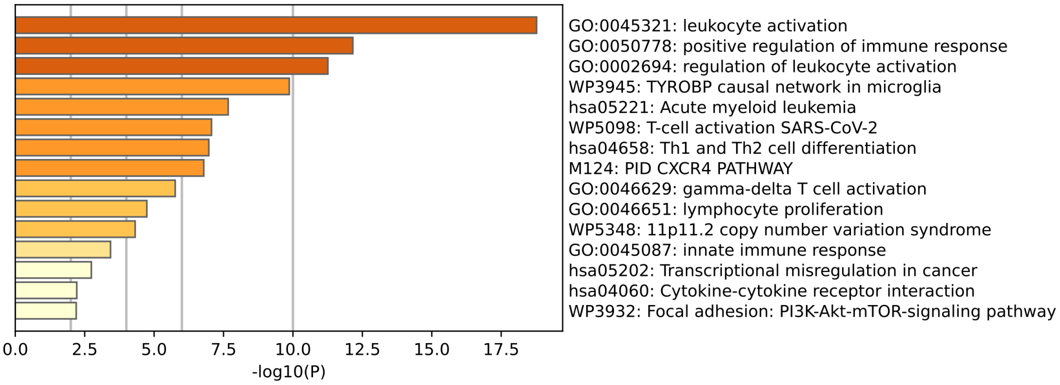

c. **ILCp: Enrichment Analysis of differentially expressed genes (*n=21*)**

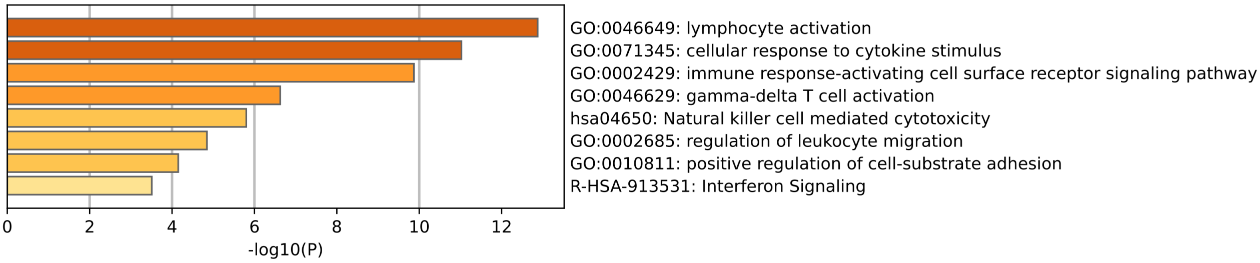

Supplementary Fig. 6. (a-c) Gene enrichment analysis of differentially expressed genes (Healthy versus COVID-19) observed in each ILCs. For each given gene list, pathway and process enrichment analysis have been carried out using metascape (default parameters) which uses the following ontology sources: KEGG Pathway, GO Biological Processes, Reactome Gene Sets, Canonical Pathways, CORUM, and WikiPathways.

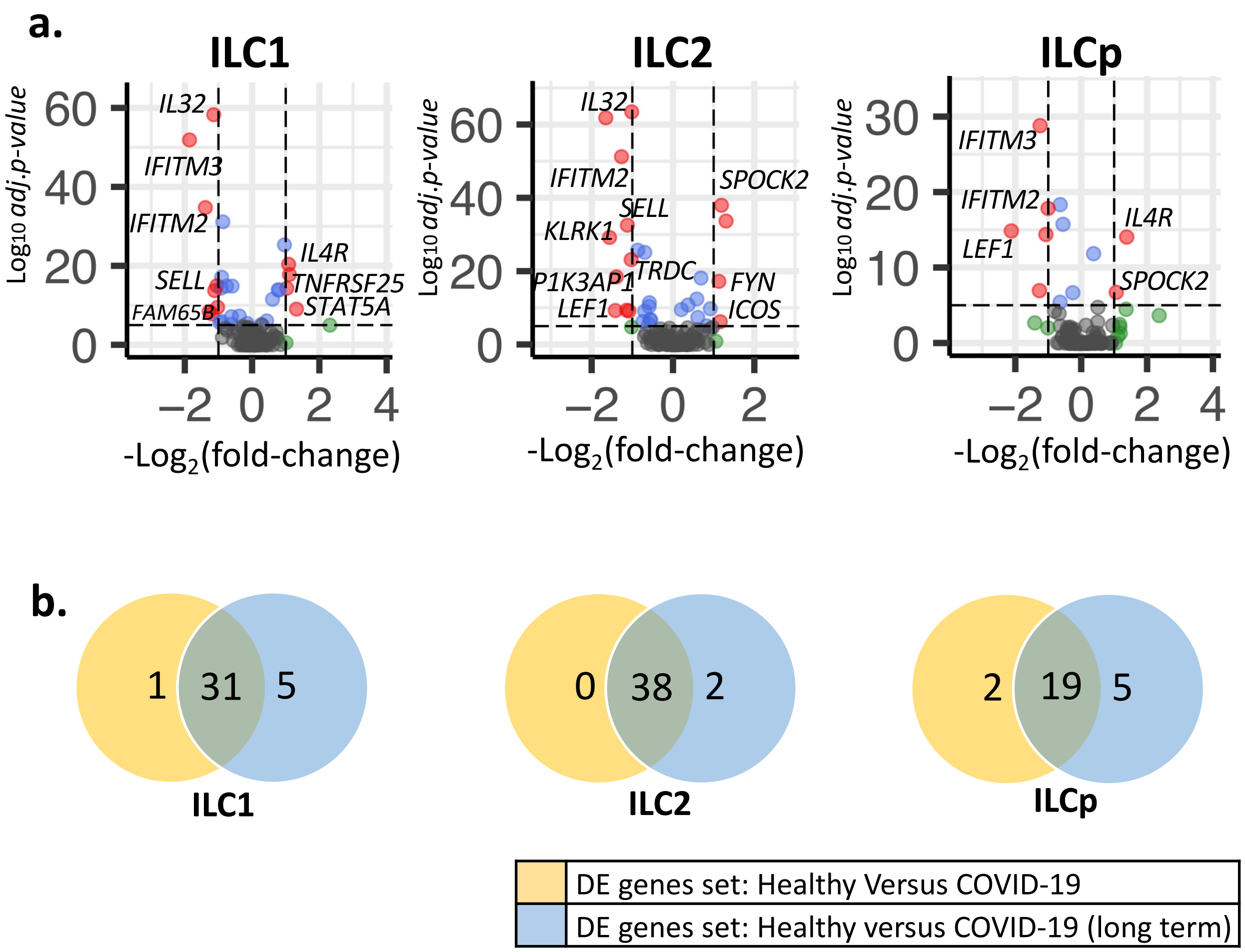

Supplementary Fig. 7. (a) Volcano plot highlighting differentially expressed genes (Healthy versus COVID-19 patients with long-term symptoms) observed in each ILCs (ILC1/ILC2/ILCp). (**b.**) The number of differentially expressed genes common between two different analyses, i.e. Healthy Versus COVID-19 and Healthy versus COVID-19 (long term symptoms).

a. **ILC1: Enrichment Analysis of differentially expressed genes (*n=36*)**

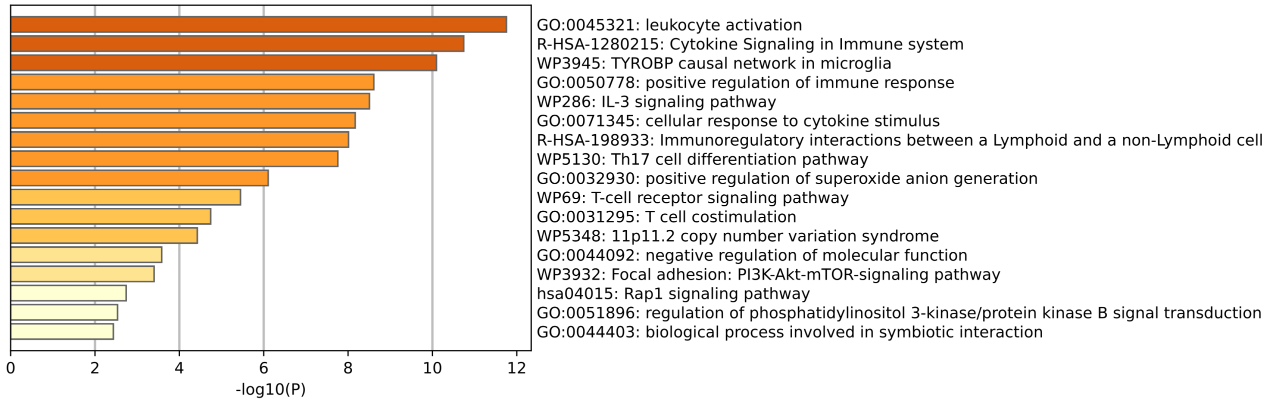

b. **ILC2: Enrichment Analysis of differentially expressed genes (*n=40*)**

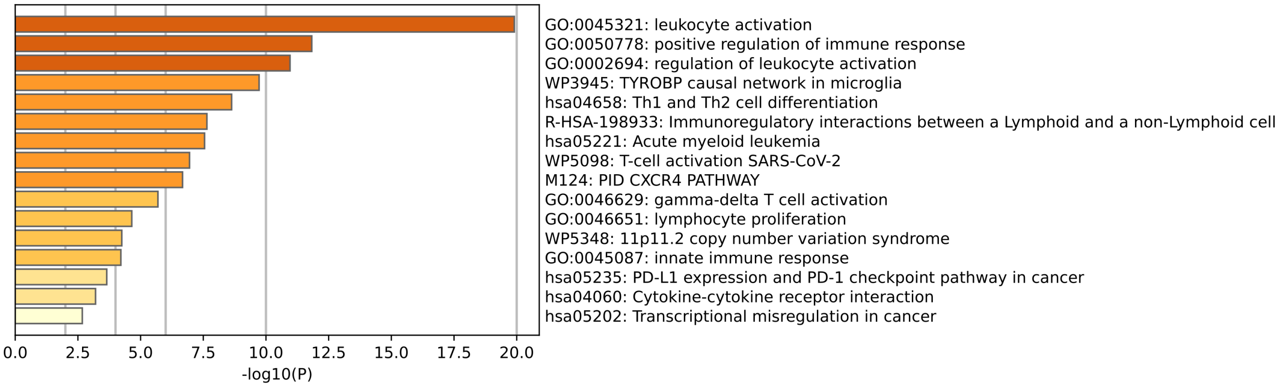

c. **ILCp: Enrichment Analysis of differentially expressed genes (*n=24*)**

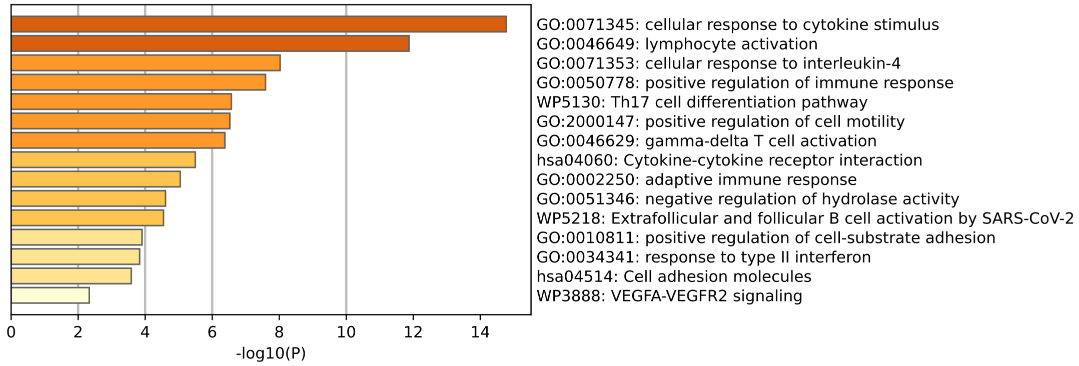

Supplementary Fig. 8. (a-c) Gene enrichment analysis of differentially expressed genes (Healthy versus COVID-19 patients with long-term symptoms) observed in each ILCs. For each given gene list, pathway and process enrichment analysis have been carried out using metascape (default parameters) which uses the following ontology sources: KEGG Pathway, GO Biological Processes, Reactome Gene Sets, Canonical Pathways, CORUM, and WikiPathways.
