# Supplementary material for: Single cell multi-omic analysis identifies key genes differentially expressed in innate lymphoid cells from COVID-19 patients
Source: Front Immunol. 2024 Jul 4;15:1374828. doi: 10.3389/fimmu.2024.1374828 (PMC11255397; doi:10.3389/fimmu.2024.1374828)
Supplement: Supplementary file 3 [file Table_1.docx]

|  | | |
| --- | --- | --- |
| **Table S1. Participant demographics.** | | |
|  | **COVID (n=22)** | **Healthy (n=25)** |
| Age in years, median (range) | 46 (+/- 22) | 46 (+/- 24) |
| Male sex, % | 54% | 23% |
| Ethnicity, % |  | |
| Hispanic | 14% | 8% |
| Race % |  | |
| AAPI^*^ | 29% | 24% |
| Afro American | 0% | 0% |
| Caucasian | 52% | 56% |
| Other | 5% | 4% |
| Days since diagnosis of COVID-19 by CLIA approved RT-PCR | 40 +/- 31.9 | N/A |
| NIH category of severity at time of diagnosis | Asymptomatic: 2  Mild: 17  Moderate: 3 | N/A |
| Long term COVID | N=15 | N/A |
| Any other conditions or illnesses? | no | no |
| Any medications during blood draw? | no | no |
| * AAPI: Asian American Pacific Islander | | |

| **Table S2. Fluorochrome-conjugated antibodies used.** | | | |
| --- | --- | --- | --- |
| **Marker** | **Fluorochrome** | **Clone** | **Manufacturer** |
| CD1a | FITC | HI149 | Biolegend |
| CD3 | FITC | HIT3a | Biolegend |
| CD4 | FITC | OKT4 | Biolegend |
| CD8 | FITC | SK1 | Biolegend |
| CD11c | FITC | ﻿Bu15 | Biolegend |
| CD14 | FITC | M5E2 | BDbiosciences |
| CD16 | FITC | 3G8 | Biolegend |
| CD19 | FITC | HIB19 | BDbiosciences |
| CD33 | FITC | HIM3-4 | Biolegend |
| CD34 | FITC | 561 | Biolegend |
| CD123 | FITC | 6H6 | Biolegend |
| CD94 | FITC | DX22 | Biolegend |
| CD303 | FITC | 201A | Biolegend |
| TCRab | FITC | IP26 | Biolegend |
| TCRgd | FITC | B1 | Biolegend |
| FcεRIα | FITC | AER-37 | Biolegend |
| CD45 | APC-Cy7 | HI30 | Biolegend |
| CD127 | PE | A019D5 | Biolegend |
| CD161 | BV421 | HP-3G10 | Biolegend |
| LIVE/DEAD FIX AQUA - 400 |  | L-34966 | Life Technologies |

| **Table S3**. Number of genes expressed (expression > 0) by ILC subsets (Median; range). | | | | |
| --- | --- | --- | --- | --- |
|  | **Healthy** | | **COVID-19** | |
|  | # Cells | Genes (median; range) | # Cells | Genes (median; range) |
| ILC1 | 918 | 45 (16-78) | 1185 | 40 (16-97) |
| ILC2 | 831 | 45 (16-86) | 1678 | 40 (16-96) |
| ILCp | 571 | 45 (16-86) | 1064 | 42 (16-93) |

| **Table S4: AbSeq oligonucleotide-linked antibodies utilized** | | | |
| --- | --- | --- | --- |
| **Marker** | **SeqID** | **Barcode Sequence** | **Manufacturer** |
| CD1a | AHS0067 | TTGGTTGCAGTGCGGTCGAAGATACGTAGTGAGATT | BD bioscience |
| CD3 | AHS0231 | AGCTAGGTGTTATCGGCAAGTTGTACGGTGAAGTCG | BD bioscience |
| CD4 | AHS0032 | TCGGTGTTATGAGTAGGTCGTCGTGCGGTTTGATGT | BD bioscience |
| CD8 | AHS0203 | AGGTGTTGGGCGGGATTGAATTCTCGTTAAGGTACT | BD bioscience |
| CD11c | AHS0183 | GTCGGTTCGTGATTTAGTTAGTGCGTCTTAGTGTCC | BD bioscience |
| CD14 | AHS0037 | TGGCCCGTGGTAGCGCAATGTGAGATCGTAATAAGT | BD bioscience |
| CD16 | AHS0242 | GCGTTTGTAGTAAGGAGATCTGCGAATAGCGTAGGG | BD bioscience |
| CD19 | AHS0030 | TAGTAATGTGTTCGTAGCCGGTAATAATCTTCGTGG | BD bioscience |
| CD33 | AHS0171 | TTCTGGGCGTTGGTAGTATTAGCGATGTATGGCGGT | BD bioscience |
| CD34 | AHS0182 | ATGGTGGCTAGGAATTGCGCTGACGTATGTTTGGGT | BD bioscience |
| CD94 | AHS0085 | GAGGTTAGGATAGGTGTACGGGTCGAGTTGAATTCT | BD bioscience |
| CD123 | AHS0020 | ACAGTTTAGTAGGACGTGAGGTATCGCGAGAATGCC | BD bioscience |
| CD303 | AHS0204 | TAAGGTAGGCAGTAGATAACGGGACGAATGATGAGC | BD bioscience |
| TCRab | AHS0078 | TTGCGTCGGATTATTAGTTCGGGTATTATGCGGTGC | BD bioscience |
| TCRgd | AHS0015 | GATTCTTATAGTCGTTGCGTAGGTTCGTCTGTGAGT | BD bioscience |
| FcεRIα | AHS0129 | GATATGGCGTGATGGTAGGTTCGGTTTAAGTTAGCG | BD bioscience |
| CD127 | AHS0028 | AGTTATTAGGCTCGTAGGTATGTTTAGGTTATCGCG | BD bioscience |
| CD161 | AHS0002 | GTTATGGTTGTCGGTAGAGTATCGTGTTGCGTTAGT | BD bioscience |
| CD117 | AHS0064 | GGATTAGTTGTCGTTATAGGGAGTGCGTTCTTAGCG | BD bioscience |
| CD45 | AHS0040 | GTGCGAAATGGCGGAATGTTATCTGCGAATGTAGTC | BD bioscience |
| CD336 | AHS0090 | AATGCAAACGATATCACGAAGGGTAGTACACGACGG | BD bioscience |
| CD294 | AHS0106 | TTAGAGTTCGTGAGAGGGTAGATCGCGTTTGTAGCC | BD bioscience |
| CD56 | AHS0257 | TGTCGGCGCGTAGTGAAGTCGTTATAATGCAGAGGT | BD bioscience |
| CD69 | AHS0010 | CAATAACGGGTCATAGTAAGTCGCGAGTAAGAGGGC | BD bioscience |
| CD45RA | AHS0009 | AAGCGATTGCGAAGGGTTAGTCAGTACGTTATGTTG | BD bioscience |
| HLA-DR | AHS0035 | TGTTGGTTATTCGTTAGTGCATCCGTTTGGGCGTGG | BD bioscience |
| CD62L | AHS0049 | ATGGTAAATATGGGCGAATGCGGGTTGTGCTAAAGT | BD bioscience |
| PD-1 | AHS0190 | ACGAGAAATGCGCGGAATGGGTGAGTTAGTAAGACG | BD bioscience |
| NKG2D | AHS0065 | TTGAAATGCGATGAGACGTAGAGCGATGTAGGTAGC | BD bioscience |

| **Table S5. BD Rhapsody extra panel.** | |
| --- | --- |
| **Gene** | **Transcript** |
| GITR (TNFRSF18) | NM_004195.2 |
| CASP3 | NM_004346.3 |
| CCNB1 | NM_031966.3 |
| CD40LG | NM_000074.2 |
| CLEC2D | NM_013269.5 |
| FBXO22 | NM_012170.3 |
| FOXO3 | NM_001455.3 |
| GATA3 | ENST00000346208.3 |
| IL6R | NM_000565.3 |
| IL9R | NM_002186.2 |
| ITK | NM_005546.3 |
| OAS1 | NM_016816.3 |
| PECAM1 | NM_000442.4 |
| PYCR1 | NM_006907.3 |
| SEMA7A | NM_003612.4 |
| SLAMF1 | ENST00000302035.10 |
| SPOCK2 | NM_014767.2 |
| TK1 | NM_003258.4 |
| TNFRSF1B | NM_001066.2 |
| XCL1 | NM_002995.2 |
| HLA-DRB3 | NM_022555.3 |
| HMGB2 | NM_002129.3 |
| IER5 | NM_016545.4 |
| CD49b | NM_002203.4 |
| CCR6 (CD196) | NM_004367.5, NM_031409.3 |
| GPR15 | NM_005290.4 |
| ITGB7 | NM_000889.3 |
| IL26 | NM_018402.2 |
| HPGDS | NM_014485.3 |
| SERPINE1 | NM_000602.5 NM_001165413.2 |
| CXCL12 | NM_000609.7 NM_001033886.2 NM_001178134.2 NM_001277990.2 NM_199168.4 |
| BDNF | NM_001143805.1 NM_001143806.1 NM_001143807.2 NM_001143808.1 NM_001143809.1 NM_001143810.1NM_001143811.1NM_001143812.1NM_001143813.2NM_001143814.2NM_001143815.1NM_001143816.1NM_001709.5NM_170731.5NM_170732.4NM_170733.3NM_170734.3NM_170735.5 |
| IL12B | NM_002187.3 |
